# Three New Species and Five New Host Records from *Chaetomiaceae* with Anti-Phytopathogenic Potential from Cover Crops *Astragalus sinicus* and *Vicia villosa*

**DOI:** 10.3390/jof10110776

**Published:** 2024-11-08

**Authors:** Ning Qian, Yuhong Wu, Wei Zhang, Jun Yang, Vijai Bhadauria, Guozhen Zhang, Jiye Yan, Wensheng Zhao

**Affiliations:** 1 MARA Key Laboratory of Surveillance and Management for Plant Quarantine Pests, Department of Plant Biosecurity, China Agricultural University, Beijing 100193, China; sy20183192472@cau.edu.cn (N.Q.); wu1507101042@126.com (Y.W.); yangj@cau.edu.cn (J.Y.); 2 Beijing Key Laboratory of Environment Friendly Management on Fruit Diseases and Pests in North China, Institute of Plant Protection, Beijing Academy of Agriculture and Forestry Sciences, Beijing 100097, China; jiyeyan@vip.163.com; 3 MARA Key Laboratory of Pest Monitoring and Green Management, Department of Plant Pathology, China Agricultural University, Beijing 100193, China; vijai.bhadauria@cau.edu.cn (V.B.); zhanggz@cau.edu.cn (G.Z.)

**Keywords:** anti-phytopathogenic potential, *Chaetomiaceae*, multigene phylogeny, morphological characteristics, new taxa

## Abstract

Cover crops, typically planted during off-seasons and requiring less agronomic manipulation, may provide abundant fungal resources. Certain species of *Chaetomiaceae* could serve as potential agents for controlling plant diseases and developing bioorganic fertilizers. Eight species from five genera of *Chaetomiaceae* were identified from healthy *Astragalus sinicus* and *Vicia villosa*, two major cover crops, through multigene phylogenetic analysis, morphological identification, and pairwise homoplasy index testing. The identified species comprise three new species: *Achaetomium astragali*, *Subramaniula henanensis*, and *S. sichuanensis*, as well as five known but new host record species: *Botryotrichum murorum*, *Chaetomium coarctatum*, *C. pseudocochliodes*, *C. pseudoglobosum*, and *Collariella pachypodioides*. Dual culture tests revealed that isolates of all eight *Chaetomiaceae* species exhibited antagonistic effects on multiple phytopathogens. Among the identified fungi, the NSJA2 isolate, belonging to *C. coarctatum*, exhibited significant relative inhibition effects on 14 out of 15 phytopathogens tested in this study, indicating its broad-spectrum antagonistic effects. Additionally, NSJA2 exhibited excellent salt tolerance. Overall, our study has identified multiple fungi with anti-phytopathogens potential, among which NSJA2 exhibits high potential for practical application. This finding paves the way for further exploration and exploitation of NSJA2 as a promising biocontrol agent.

## 1. Introduction

The family *Chaetomiaceae*, belonging to the order *Sordariales* in the class *Sordariomycetes* of Ascomycota, is known for its diverse range of fungi that produce non-stromatic ascomata with specific morphological characteristics. The family comprises 50 genera and 275 species that exhibit high phenotypical and ecological diversity [1]. Some species within *Chaetomiaceae* have gained attention due to their potential biotechnological applications. For instance, *Chaetomium globosum* Kunze ATCC 16,021 has been found to possess the ability to degrade certain polymers [2], while *Myceliophthora thermophila* (Apinis) Oorschot has been utilized for its secretion of various hydrolytic enzymes, with applications in biotechnology [3].

In agricultural systems, *Chaetomiaceae* fungi are being explored as potential biocontrol agents for managing plant diseases and as bioorganic fertilizers to promote plant growth. Research has shown that these fungi can improve soil microbial community structure and metabolic activity in cotton plants, thereby aiding in the prevention of wilt disease and promoting growth [4]. *Humicola phialophoroides* W.H. Koet al. could produce various resistance-activating substances for controlling Phytophthora blight of pepper [5]. The genus *Chaetomium*, particularly the species *C. globosum*, has been extensively studied within the family of *Chaetomiaceae*. *Chaetomium globosum* has been utilized as a biological control agent to manage sclerotinia disease in rape and tan spot disease in wheat [6,7]. Moreover, it has demonstrated the ability to inhibit various phytopathogens, including species of *Alternaria*, *Colletotrichum*, *Fusarium*, and *Phytophthora* [4,8,9,10,11,12,13,14,15,16]. *Chaetomium globosum* is recognized as a prolific producer of secondary metabolites for controlling plant diseases, with over 300 active secondary metabolites isolated from it to date. Chaetoglobosins and Azaphilones are the main types of secondary metabolites found in *Chaetomium* spp., and they have been proven to have multiple functions. In the prevention and control of plant diseases, it has been found that they have inhibitory activity against many plant pathogens [17,18,19,20,21,22]. Chaetoglobosin A, a type of Chaetoglobosins, and Chaetoviridin A, a type of Azaphilones, both exhibit broad-spectrum antagonistic activity against numerous phytopathogens [23]. Additionally, Chaetoglobosin A has shown strong nematicidal activity [24,25].

In China, *Astragalus sinicus* and *Vicia villosa* are two cover/green manure crops from *Fabaceae* with a long history of cultivation and utilization [26]. Although most cover crops are planted in a conservation tillage mode of crop rotation or intercropping with the main crop, little attention has been paid to the fungal diversity on such crops. Recently, we investigated the fungal diversity on *A. sinicus* and *V. villosa*, describing seven new species and twenty-four new host records from these plants [26]. Further microbiome analysis revealed many unexplored fungal communities with excellent potential application prospects on *A. sinicus* and *V. villosa* [26]. In this study, ten isolates of *Chaetomiaceae*, belonging to eight species in five genera, were isolated from healthy *A. sinicus* and *V. villosa* plants collected from the Guangxi, Henan, and Sichuan Provinces, China. All the isolates exhibited broad-spectrum antagonistic activity. Among them, the NSJA2 isolate showed significant relative inhibition effects on 14 of 15 phytopathogens. Our results reveal that the *Chaetomiaceae* species from cover crops could provide potential biocontrol resources.

## 2. Materials and Methods

### 2.1. Sample Collection, Fungal Isolation, and Preservation

Healthy leaf and stem samples of *A. sinicus* and *V. villosa* were collected from fields in Xinyang City (Henan Province, China), Nanchong City (Sichuan Province, China), and Nanning City (Guangxi Province, China), in 2020 and 2021. The fungi were isolated and purified using the method described by Chomnunti et al. [27] and Senanayake et al. [28]. Samples were surface-sterilized by dipping in 75% ethanol for 15 s, 5% NaOCl for 3 min, and then washed three times with sterile distilled water and dried on sterilized filter paper. Tissue pieces taken from surface-sterilized samples were placed on OA (4% oatmeal, 2% agar, and distilled water) plates. The plates were incubated at 25 °C in the light/dark alternation for 10 d. Single-spore or single-hyphal purified isolates were preserved in cryotubes containing PDA medium at 4 °C. The fungal cultures were deposited at the China General Microbiological Culture Collection Center (CGMCC) and the Culture Collection of the Institute of Plant Protection, Beijing Academy of Agriculture and Forestry Sciences (JZB). The holotype of the novel species was deposited at the Fungarium (HMAS), Institute of Microbiology, Chinese Academy of Sciences (CAS).

### 2.2. DNA Extraction, PCR Amplifcation, and Sequencing

Genomic DNA was extracted using the Quick and Safe (QS) method as described by Chi et al. [29]. Four loci, including the internal transcribed spacer regions and intervening 5.8S nrRNA gene (ITS), large subunit ribosomal RNA gene (LSU) and partial sequences of the β-tubulin (*tub2*), and RNA polymerase II second largest subunit (*rpb2*) genes, were amplified and sequenced with the primers ITS5/ITS4, LR5/LROR, T1/TUB4Rd, and rpb2-5F2/rpb2-7CR, respectively [1]. The PCR amplification was carried out in 50 μL of a reaction mixture that contained 25 μL of 2 × M5 HiPer plus Taq HiFi PCR mix (with blue dye, amplification speed: 10–15 s/kb, Mei5 Biotechnology Co., Ltd., Beijing, China), 22 μL of ddH_2_O, 1 μL of each primer (10 μM, Sangon Biotech company Co., Ltd., Shanghai, China), and 1 μL of genomic DNA (about 50 ng DNA). The following PCR amplification procedures are used to amplify ITS, LSU, and *rpb2* and *tub2* gene fragments: pre-denaturation at 95 °C for 3 min (1 cycle); denaturation at 94 °C for 25 s, annealing at 55 °C for 25 s, and extension at 72 °C for 15 s (35 cycles); and then final extension at 72 °C for 5 min (1 cycle). The PCR products were checked on 1% agarose gel under UV light using Alphalmager Mini (ProteinSimple, San Jose, CA, USA) after M5 Gelred Plus Nucleic Acid Dye (Mei5 Biotechnology company, Beijing, China) staining and then sent to Tsingke Biotech Co., Ltd. (Beijing, China) for sequencing. Forward and reverse amplicon sequences were aligned with DNAMAN software (5.2.2, Lynnon Corp, Vaudreuil, QC, Canada) to obtain consensus sequences, which were submitted to GenBank. The accession numbers for the reference sequences of ITS, LSU, *rpb2*, and *tub2* obtained from GenBank [1] are listed in Table S1.

### 2.3. Phylogenetic Analyses

The individual gene datasets were aligned using MAFFT 7 with default settings [30] (http://mafft.cbrc.jp/alignment/server). The sequence alignments were carefully examined in BioEdit [31], and any errors were manually corrected. Phylogenetic analyses were conducted using three methods: maximum parsimony (MP), maximum likelihood (ML), and Bayesian inference (BI). For MP and ML, analyses were carried out using PAUP 4.0b10 with 1000 bootstrap replications [32] and RAxML-HPC2 on XSEDE (8.2.12) with the GTRGAMMA + I model and 1000 non-parametric bootstrapping iterations in the CIPRES Science Gateway platform [33], respectively. The evolutionary models for each locus used in Bayesian analysis were selected using jModelTest2 on XSEDE (2.1.6) in the CIPRES Science Gateway platform. Meanwhile, Bayesian inference analysis was carried out using MrBayes on XSEDE (3.2.7a) in the CIPRES Science Gateway platform. In BI analysis, posterior probabilities (PPs) were determined by Markov Chain Monte Carlo sampling (BMCMC), and different evolutionary models were used in response to the gene regions. For the combined dataset, six simultaneous Markov chains were run for 8,000,000 generations, and trees were sampled at every 1000th generation. The first 25% of the generated trees were discarded, and the remaining 75% of trees were used to calculate the posterior probabilities (PPs) of the majority rule consensus tree [34]. The results of each analysis were visualized with FigTree v1.4.0. The final alignment generated in this study was submitted to TreeBASE (https://treebase.org/treebase-web/home.html).

The phylogenetically related ambiguous species were analyzed using the Genealogical Concordance Phylogenetic Species Recognition (GCPSR) model with a pairwise homoplasy index (PHI) test. The PHI test was performed in SplitsTree4 to determine the recombination event within phylogenetically closely related species using a four-locus concatenated dataset (ITS, LSU, *rpb2*, *tub2*), and results with a PHI below 0.05 indicated that there was significant recombination in the dataset. The relationship between closely related species was visualized by constructing a splits graph [35].

### 2.4. Morphology

Colony morphology was determined by inoculating strains onto OA and CMA (6% cornmeal, 2% agar, and distilled water) [1,36]. The media were prepared as described by Crous et al. (2009). After incubation in the light/dark alternative at 25 °C for 7 days, colonies and ascomata were observed and photographed using a Nikon-SMA1500 dissecting microscope (Nikon, Tokyo, Japan). Shear’s mounting solution was used to observe the asci, and lactic acid was used to observe the ascomata, ascomatal hairs, and ascospores [37,38]. Morphological characteristics of the fungal cultures were observed and photographed under the Axio Imager Z2 light microscope (Carl Zeiss Microscopy, Oberkochen, Germany). ZEN PRO 2012 software (Carl Zeiss Microscopy) was employed for measurement, and Adobe Photoshop CC2019 software was used to prepare the morphological photo plates (Adobe Systems, San Jose, CA, USA). Taxonomic descriptions for novel species were deposited in Mycobank.

### 2.5. Dual Culture Test

The dual culture method described by Wang et al. [39] was employed for this study. The mycelial disks (5 mm diameter) taken from the *Chaetomiaceae* isolates obtained in this study and phytopathogens were transferred onto the border of PDA plates and incubated at 25 °C for 5–7 days. The antagonistic growth was measured using the radius of two colonies facing each other, and the relative inhibiting effects were calculated to evaluate the antagonistic ability [40]. The radial inhibited rate and relative inhibiting effect were calculated using the following formula: radial inhibited rate = (the radius of colonies of single culture − the radius of dual culture colonies)/(the radius of colonies of single culture − the radius of mycelial disks) 100%; relative inhibiting effect index = (the inhibited rate of phytopathogens)/(the inhibited rate of *Chaetomiaceae* isolates) [41].

The phytopathogens used in this study included fourteen fungi and one chromista, causing various plant diseases, such as leaf spot, canker, wilt, and rot diseases, in different crops. These include *Alternaria alternata* (A33) and *Stemphylium astragali* (XZYB6f) causing leaf spot on *A. sinicus*, *A. brassicae* (HEYA2) causing leaf spot on *Orychophragmus violaceus*, *Boeremia linicola* (Y3-3) causing leaf spot on clover, *Botryosphaeria dothidea* (B-8-1) causing ring rot on pear, *Botrytis cinerea* (B79) causing gray mold on strawberry, *Colletotrichum siamense* (CCT1) causing anthracnose on strawberry, *Fusarium graminearum* (F0609) causing wheat scab, *F. oxysporum f.* sp. *cucumerinum* (Foc) causing cucumber *fusarium* wilt, *Lasiodiplodia theobromae* (CSS-01S) causing grape canker disease, *Sclerotinia minor* (HJ5) and *S. sclerotiorum* (ZJ25) causing stem rot on *A. sinicus*, *Pyricularia oryzae* (P131) causing rice blast, *Verticillium dahliae* (V991) causing cotton wilt disease, and *Phytophthora capsici* (LT263) causing pepper blight.

### 2.6. Salt Tolerance Ability Assessment

The tolerance of the broad-spectrum antagonistic (BSA) isolate to salt was assessed in PDA culture media with varying concentrations of NaCl. This involved placing 5 mm diameter mycelial disks of the BSA isolate taken from the margin of a 5-day-old colony individually in the center of each PDA plate with NaCl. The control treatment consisted of a PDA plate inoculated with the BSA isolate but without NaCl. The experiment was repeated three times, and the plate culture conditions involved alternating light and dark at 25 °C for 7 days. The tolerance index (TI) was calculated using the formula described by Colpaert et al. [42]: TI = (colony diameter on PDA plate with NaCl − colony diameter of mycelial disks)/(colony diameter on the control PDA plate − colony diameter of mycelial disks) × 100%.

### 2.7. Statistical Analysis

The data shown represent the average values along with the standard deviations (mean ± SDs) from three independent replications. The SPSS software (SPSS Statistics, Version 17.0. SPSS, Chicago, IL, USA) was used for statistical analyses. Data were analyzed using a one-way ANOVA analysis of variance. Duncan’s multiple range test (*p* < 0.05) was used to determine significant differences between sample means [43].

## 3. Results

### 3.1. Fungal Isolates

Ten fungal strains were isolated from the leaves and stems of healthy *Astragalus sinicus* and *Vicia villosa* plants collected from three provinces in China. Based on the NCBIBLAST results of ITS and LSU sequences, we initially identified these isolates as belonging to five genera in *Chaetomiaceae* (Table 1).

### 3.2. Phylogenetic Analyses

**Figure 1:**
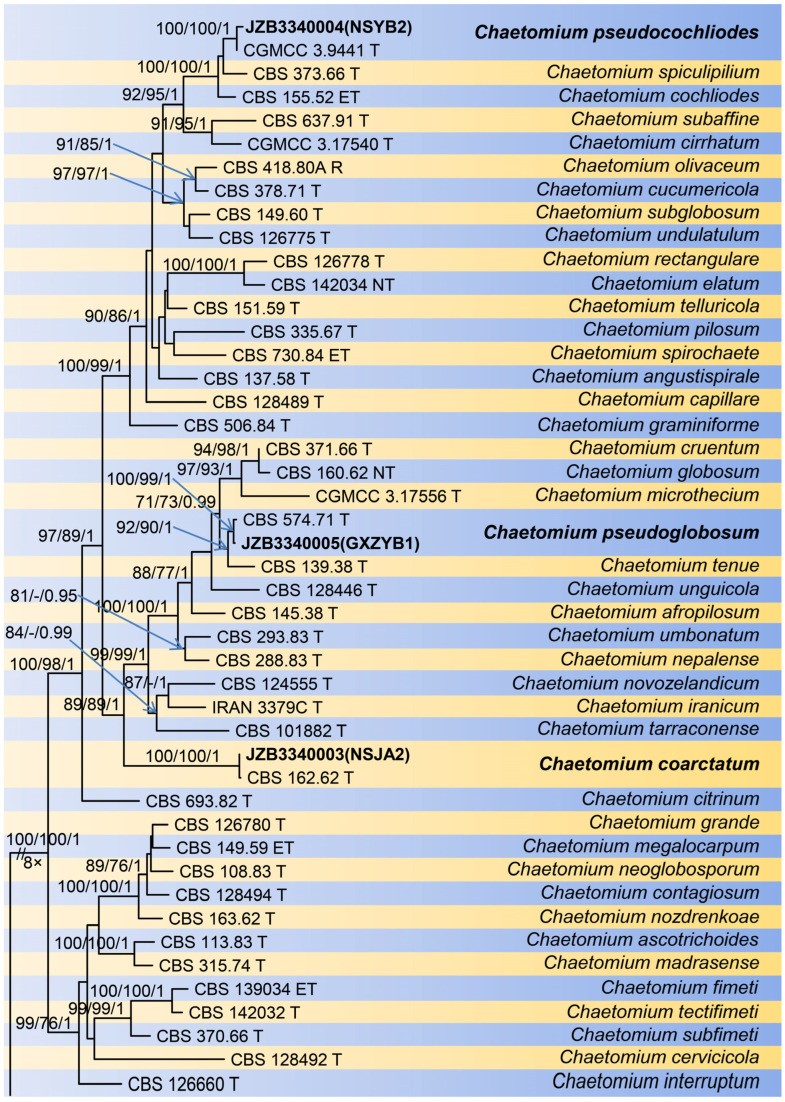

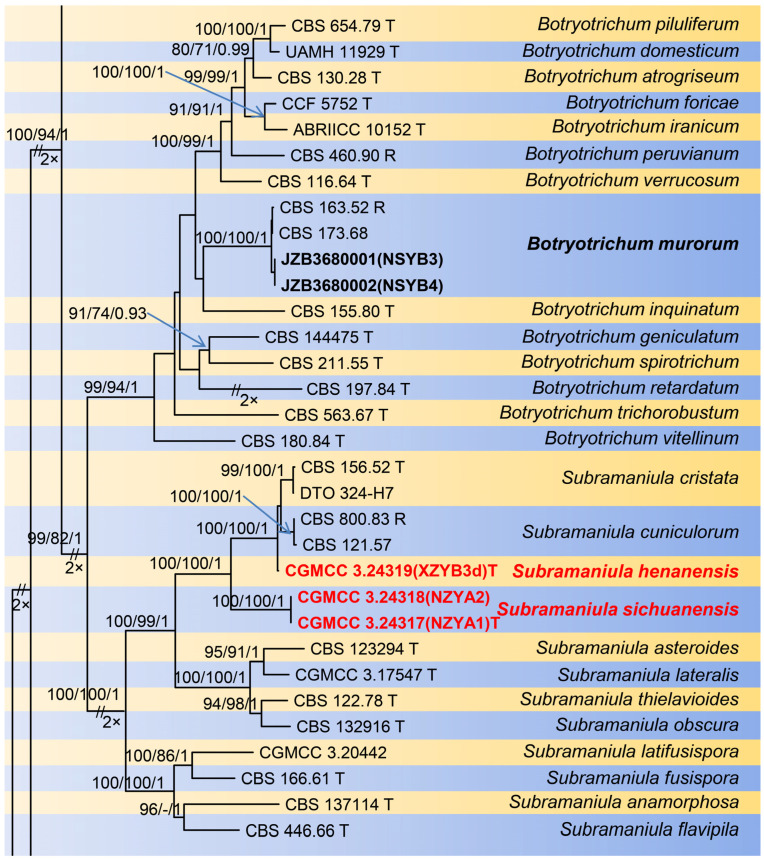

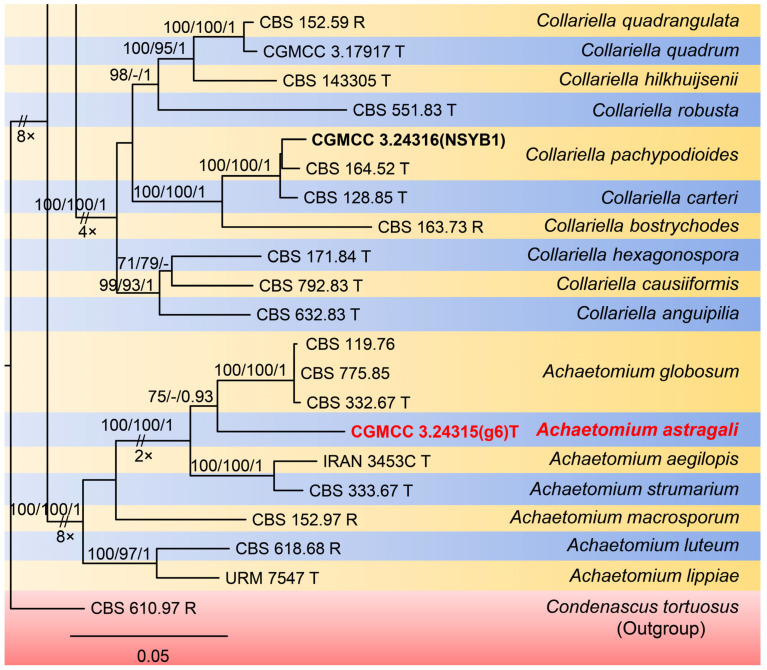
Phylogenetic tree generated from maximum likelihood analysis based on combined rpb2, tub2, ITS, LSU sequence data of five genera of *Chaetomiaceae*. The tree is rooted with *Condenascus tortuosus* (CBS 610.97). The tree topology of the maximum parsimony analysis and Bayesian posterior probability analysis is similar to the maximum likelihood analysis, so its tree topology is no longer displayed, and only the bootstrap value is added. The scale bar indicates 0.05 expected changes per site. Maximum likelihood bootstrap support (MLBS) values, maximum parsimony bootstrap support (MPBS) values ≥ 70%, and Bayesian posterior probabilities (BYPP) ≥ 0.90 are indicated on the branches. All ex-type strains are in bold. New isolates are in bold, and new species are in red.

A total of 99 strains were used to conduct multigene phylogenetic analyses, with *Condenascus tortuosus* (CBS 610.97) as an outgroup taxon. The concatenated alignment consisted of 2697 characters (including alignment gaps): 852, 748, 568, and 529 characters were used in the *rpb2*, *tub2*, ITS, and LSU partitions, respectively (Figure 1). For the MP analysis, the concatenated alignment contained 1598 constant characters, 166 variable and parsimony-uninformative characters, and 911 parsimony-informative characters. The MP analysis yielded 1000 equally most parsimonious trees (TL = 5531; CI = 0.344; RI = 0.785; RC = 0.270; HI = 0.656). For the ML analysis, the best-scoring RAxML tree with a final optimization likelihood value of −30,710.334022 is presented. The ML matrix comprised 1236 distinct alignment patterns, with 16.78% of undetermined characters or gaps. The estimated base frequencies were as follows: A = 0.228595, C = 0.283810, G = 0.281142, T = 0.206453; substitution rates AC = 1.252619, AG = 4.749008, AT = 1.398978, CG = 1.596481, CT = 7.113217, and GT = 1.000000 (gamma distribution shape parameter α = 1.118540). For the BI analysis, TrN + I + G, HKY + I + G, TIM2 + I + G and TIM3ef + I + G models were selected for the *rpb2*, *tub2*, ITS, and LSU partitions, respectively (Table 2). These models were incorporated into the analysis. The alignment generated in this study is submitted to TreeBASE under submission number 31,717.

### 3.3. Taxonomy

**Figure 2:**
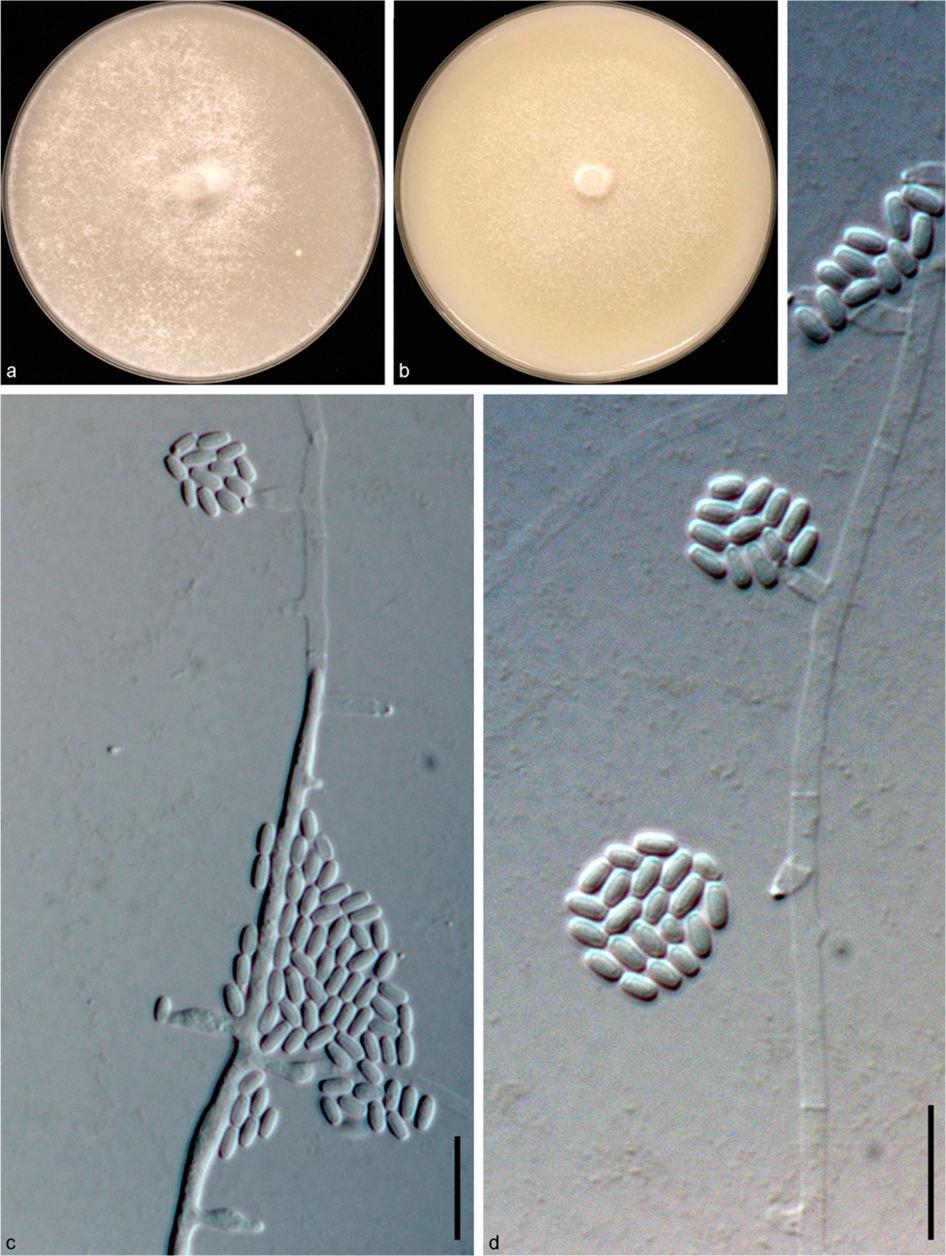
*Achaetomium astragali* sp. nov (g6/CGMCC 3.24315). (**a**,**b**) Colonies on OA (**a**) and CMA (**b**); (**c**,**d**), conidia and conidiophore. Scale bars = 10 μm.

*Achaetomium astragali* N. Qian and W. S. Zhao, sp. nov. (Figure 2).

MycoBank MB851542.

Etymology—‘astragli’ refers to the host plant genus Astragalus.

Type: CHINA: Henan Province: Xinyang, Gushi, 115°68′69.8″ E, 32°18′01.1″ N, isolated from healthy leaves of *Astragalus sinicus*, 11 Apr. 2019, W. S. Zhao (holotype HMAS 352361, ex-type culture CGMCC 3.24315 = g6).

Genbank accession numbers: ITS: PP062902, LSU: PP062912, *rpb2*: PP067881, *tub2*: PP067871.

Descriptions: Sexual morph: not observed. Asexual morph: Mycelium branched, septate, smooth, hyaline, hyphae 1–4 μm diam. Conidiophores phialidic, formed laterally from aerial hyphae, solitary, monoblastic, 3–8.8 μm long, 1.1–3 μm diam at the base. Conidia arranged in false heads on conidiogenous cell tips, hyaline, aseptate, smooth, ovate or elliptic, 2.4–4.6 × 1.5–2.1 μm (av. ± SD: 3.3 ± 0.4 × 1.8 ± 0.1 μm).

Cultural characteristics: Colonies on OA reaching 90 mm in diameter after 5 d at 25 °C, circular, flat, felty, at first white, becoming bright pink. Colonies on CMA reaching 90 mm in diameter after 7 d at 25 °C, circular with smooth margin, flat, hyphae grow faintly and loosely.

**Figure 3:**
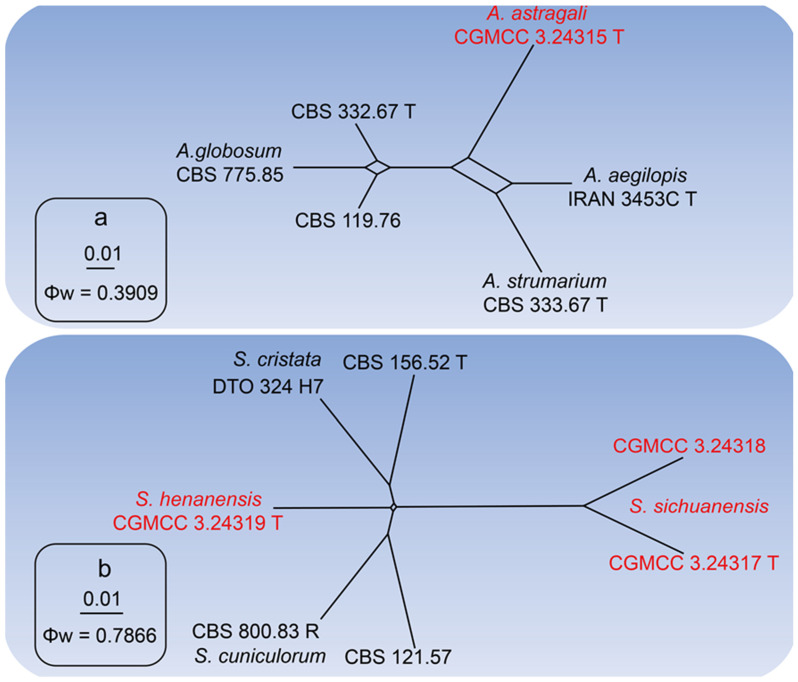
The result of the pairwise homoplasy index (PHI) tests of closely related species using both LogDet transformation and Splits decomposition. The PHI of *Achaetomium astragali* (**a**) or *Subramaniula henanensis*, *Subramaniula sichuanensis* (**b**) and their phylogenetically related isolates or species, respectively. PHI test value (Φw) < 0.05 indicates significant recombination within the dataset. New species and their ex-type cultures are indicated with red color.

Notes: In this study, we obtained an isolate (CGMCC 3.24315) from healthy leaves of *A. sinicus*, and based on multi-loci phylogenetic analysis, this isolate formed a sister clade to reference strains of *Achaetomium globosum* (CBS 119.76, CBS 775.85, and CBS 332.67T) with high statistical support (MLBS: 75%, MPBS: 65%, BYPP: 0.93). When comparing the nucleotide differences of our isolate with the type strain of *A. globosum* (CBS 332.67T), we observe nucleotide differences in all gene regions as follows; ITS: 7%, LSU: 2%, *rpb2*: 10%, and *tub2*: 9%. Furthermore, the PHI test (w = 0.3909) did not detect any recombination events between our isolate (CGMCC 3.24315) and other *Achaetomium* species (Figure 3a).

In this study, our *Achaetomium* isolate showed asexual morph characters, and so far, all the other species in the *Achaetomium* genus reported only sexual morph characters. This genus is characterized by having superficial ascocarps, outgrowth hairs of devoid, and ostiolate and 8-spored asci, clavate, without any apical structures. Further species of *Achaetomium* have colored ascospores without any appendages, which are globose to elliptical, compressed or simple, and with one to two polar pores [44]. Therefore, in this study, we were not able to compare the morphological characteristics of our isolate (CGMCC 3.24315) with other existing *Achaetomium* species, and we could not link the sexual–asexual morph of *Achaetomium* due to the insufficient data.

Therefore, here, based on phylogenetic evidence, we would like to keep our isolate as a novel species, *Achaetomium astragali* sp. nov., for now, and we would like to recommend an urgent generic revision of *Achaetomium* with re-collection or novel species collections, and as new data emerge, we hope the asexual–sexual link of this genus will resolve.

**Figure 4:**
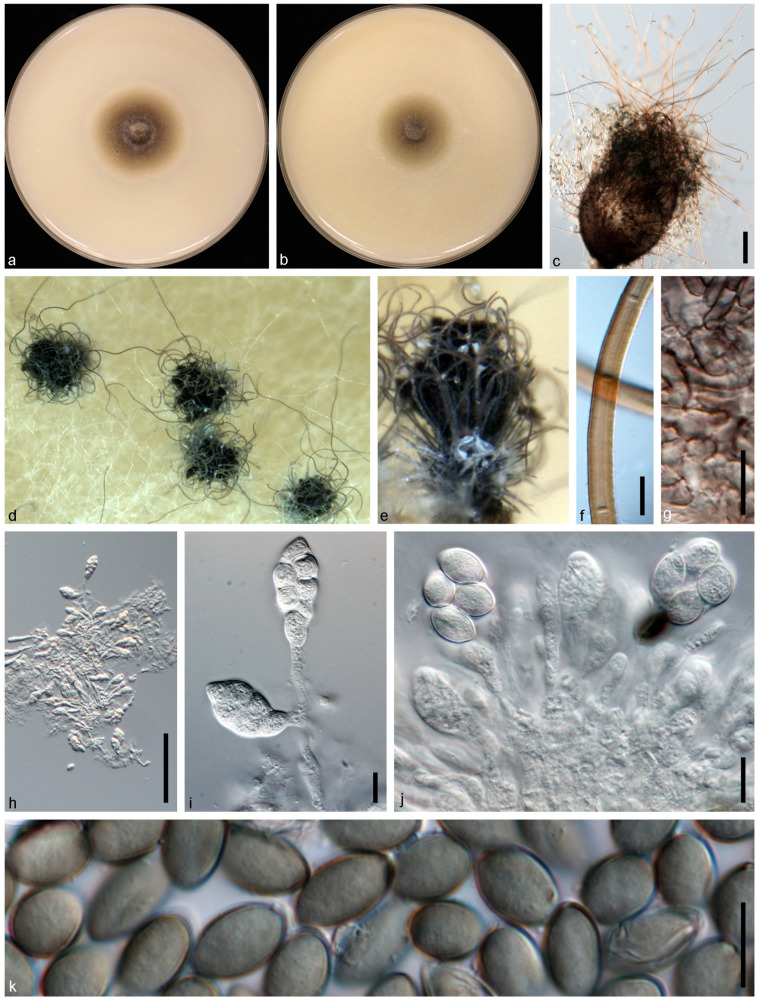
*Botryotrichum murorum* (JZB3680002/NSYB4). (**a**,**b**) Colonies on OA (**a**) and CMA (**b**); (**c**) ascomata mounted in lactic acid; (**d**,**e**) mature ascomata on OA, top view (**d**) and side view (**e**); (**f**) terminal hairs; (**g**) ascomatal wall; (**h**–**j**) asci; (**k**) ascospores. Scale bars: (**f**,**g**,**i**–**k**) = 10 μm; (**c**,**h**) = 100 μm.

*Botryotrichum murorum* (Corda) X.W. Wang and Samson, Stud. Mycol. 84: 164. 2016. (Figure 4).

Description and Illustration—Wang et al. [37].

Material examined: CHINA: Sichuan Province: Guangan, Yuechi, 106°44′07.9″ E, 30°53′91.8″ N, from healthy leaves of *Vicia villosa*, 19 Mar. 2020, Y.H. Wu (dry culture HMAS 352366; HMAS 352367, living cultures JZB3680001 = NSYB3; JZB3680002 = NSYB4).

Genbank accession number: JZB3680001 = ITS: PP062900, LSU: PP062910, rpb2: PP067879, tub2: PP067869; JZB3680002 = ITS: PP062901, LSU: PP062911, *rpb2*: PP067880, *tub2*: PP067870.

Notes: In this study, we obtained two *Botryotrichum isolates* (JZB3680001, JZB3680002) from healthy leaves of *V. villosa*, and based on morphological and multi-loci phylogenetic analyses, we identified them as *Botryotrichum murorum*. In a multi-loci phylogenetic tree, our two isolates are in a clade with the reference isolates of *B. murorum* (CBS 163.52R and CBS 173.68) with high statistical support (MLBS: 100%/MPBS: 100%/BYPP: 1.00) (Figure 1). When comparing the nucleotide differences of our isolates with the reference isolate of *B. murorum* (CBS 163.52R), we observe high nucleotide similarity in all gene regions (ITS: 100%, LSU: 100%, *rpb2*: 100%, and *tub2*: 99.4%). To the best of our knowledge, this is the first report of *B. murorum* associated with *Vicia villosa* worldwide.

**Figure 5:**
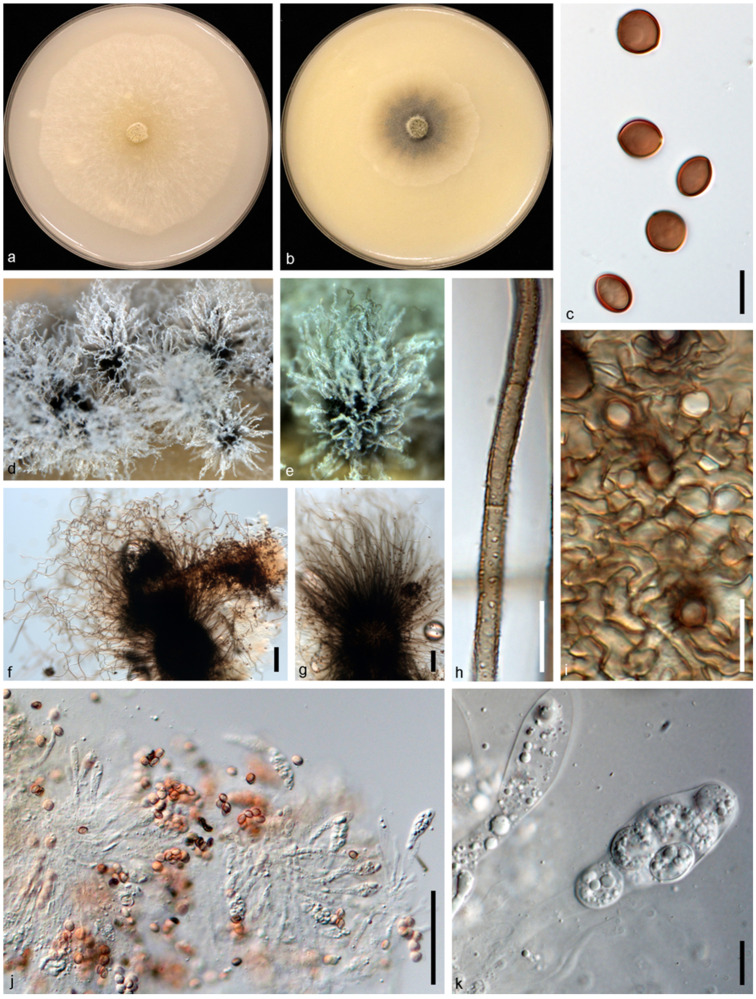
*Chaetomium coarctatum* (JZB 3,340,003/NSJA2). (**a**,**b**) Colonies on OA (**a**) and CMA (**b**); (**c**) ascospores; (**d**,**e**) mature ascomata on OA, top view (**d**) and side view (**e**); (**f**,**g**) ascomata mounted in lactic acid; (**h**) terminal hairs; (**i**) ascomatal wall; (**j**,**k**) asci. Scale bars: (**c**,**h**,**i**,**k**) = 10 μm; (**f**,**g**,**j**) = 100 μm.

*Chaetomium coarctatum* Sergeeva, Not. Syst. sect. Crypt. Inst. Bot. Acad. Sci. U.S.S.R. 14: 146. 1961. (Figure 5).

Description and Illustration—Wang et al. [38].

Material examined: CHINA: Sichuan Province: Guangan, Yuechi, 106°44′07.9″ E, 30°53′91.8″ N, from healthy stems of *Vicia villosa*, 19 Mar. 2020, Y.H. Wu (dry culture HMAS 352362), living culture JZB 3,340,003 = NSJA2).

Genbank accession number: ITS: PP062897, LSU: PP062907, *rpb2*: PP067876, *tub2*: PP067866.

Notes: In this study, we obtained an isolate (JZB 3340003) from healthy stems of *V. villosa* that showed morphological similarity to the *Chaetomium* species, and based on both morpho-molecular data, it has been identified as *Chaetomium coarctatum*. In multi-locus phylogeny, our isolate is in a clade with the ex-type strain of *C. coarctatum* (CBS 162.62), with a high bootstrap support (MLBS: 100%; MPBS: 100%; BYPP: 1.00). When comparing the nucleotide differences of our isolate with the ex-type strain of *C. coarctatum* (CBS 162.62), we observe high nucleotide similarity in four loci (ITS: 99.8%, LSU: 100%, *rpb2*: 100%, *tub2*: 100%). To the best of our knowledge, this is the first report of *C. coarctatum* associated with *V. villosa* worldwide.

**Figure 6:**
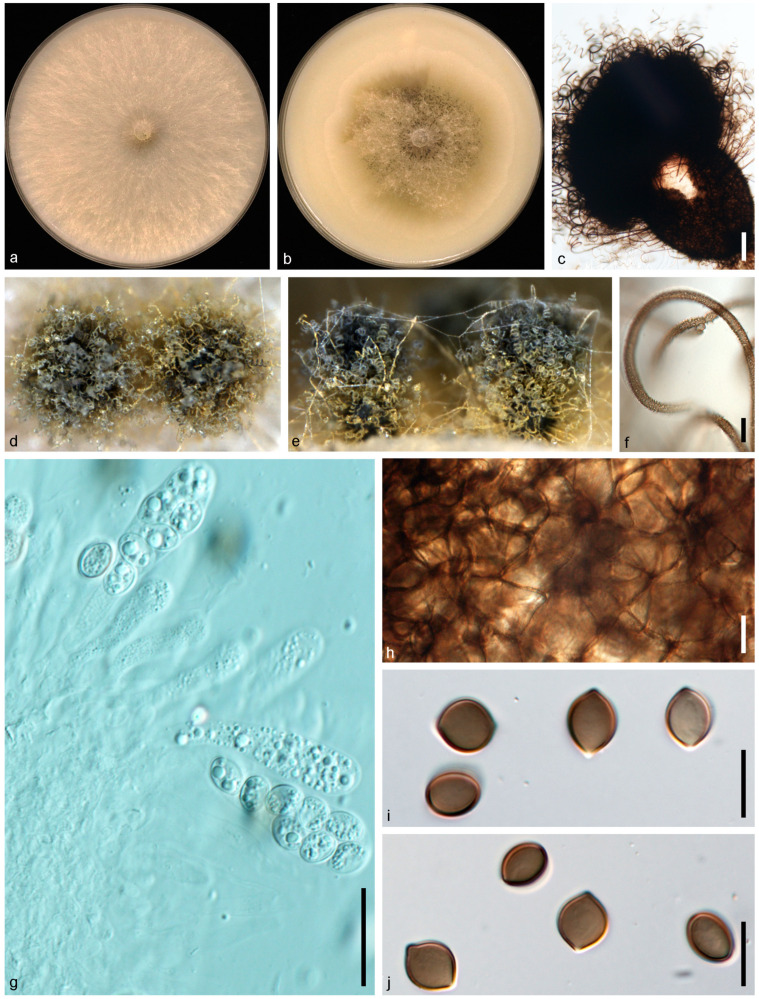
*Chaetomium pseudocochliodes* (JZB3340004/NSYB2). (**a**,**b**) Colonies on OA (**a**) and CMA (**b**); (**c**) ascomata mounted in lactic acid; (**d**,**e**) mature ascomata on OA, top view (**d**) and side view (**e**); (**f**) terminal hairs; (**g**) asci; (**h**) ascomatal wall; (**i**,**j**) ascospores. Scale bars: (**f**,**h**–**j**) = 10 μm, (**g**) = 20 μm, (**c**) = 100 μm.

*Chaetomium pseudocochliodes* X.W. Wang, X.Z. Liu and Crous, Persoonia 36: 113. 2016. (Figure 6).

Description and Illustration—Wang et al. [38].

Material examined: CHINA: Sichuan Province: Guangan, Yuechi, 106°44′07.9″ E, 30°53′91.8″ N, from healthy leaves of *Vicia villosa*, 19 Mar. 2020, Y.H. Wu (dry culture HMAS 352363, living culture JZB3340004 = NSYB2).

Genbank accession number: ITS: PP062899, LSU: PP062909, *rpb2*: PP067878, *tub2*: PP067868.

Notes: In this study, we obtained an isolate (JZB3340004) from healthy leaves of *V. villosa* that showed morphological similarity to the Chaetomium species, and based on both morpho-molecular data, it has been identified as *Chaetomium pseudocochliodes*. In multi-locus phylogeny, our isolate is in a clade with the ex-type strain of *C. pseudocochliodes* (CGMCC 3.9441), with a high bootstrap support (MLBS: 100%; MPBS: 100%; BYPP: 1.00). When comparing the nucleotide differences of our isolate with the ex-type strain of *C. pseudocochliodes* (CGMCC 3.9441), we observed high nucleotide similarity in four loci (ITS: 99.6%, LSU: 100%, *rpb2*: 99.8%, *tub2*: 99.8%). To the best of our knowledge, in this study, we are the first to report the novel host association of *C. pseudocochliodes* on *V. villosa* worldwide.

**Figure 7:**
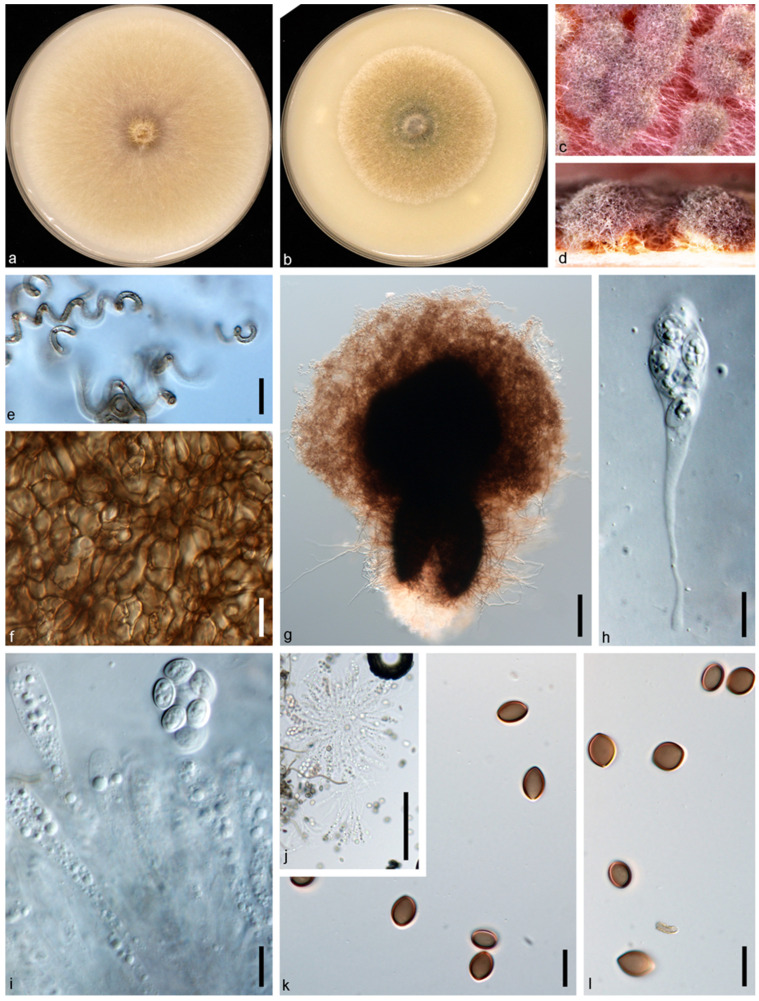
*Chaetomium pseudoglobosum* (JZB3340005/GXZYB1). (**a**,**b**) Colonies on OA (**a**) and CMA (**b**); (**c**,**d**) mature ascomata on OA, top view (**c**) and side view (**d**); (**e**) terminal hairs; (**f**) ascomatal wall; (**g**) ascomata mounted in lactic acid; (**h**–**j**) asci; (**k**,**l**) ascospores. Scale bars: (**e**,**f**,**h**,**i**,**k**,**l**) = 10 μm; (**g**,**j**) = 100 μm.

*Chaetomium pseudoglobosum* X.W. Wang, X.Z. Liu and Crous, Persoonia 36: 115. 2016. (Figure 7).

Description and Illustration—Wang et al. [38].

Material examined: CHINA: Guangxi Province: Nanning, Longan, 107°69′19.2″ E, 23°17′33.6″ N, from healthy leaves of *Astragalus sinicus*, 14 Mar. 2020, W.S. Zhao (dry culture HMAS 352364, living culture JZB3340005 = GXZYB1).

Genbank accession number: ITS: PP062903, LSU: PP062913, *rpb2*: PP067882, *tub2*: PP067872.

Notes: In this study, we obtained an isolate (JZB3340005) from healthy leaves of *A. sinicus* that showed morphological similarity to the *Chaetomium* species, and based on both morpho-molecular data, it has been identified as *Chaetomium pseudoglobosum*. In multi-locus phylogeny, our isolate is in a clade with the ex-type strain of *C. pseudoglobosum* (CBS 574.71), with a high bootstrap support (MLBS: 100%; MPBS: 99%; BYPP: 1.00). When comparing the nucleotide differences of our isolate with the ex-type strain of *C. pseudoglobosum* (CBS 574.71), we observe high nucleotide similarity in four loci (ITS: 100%, LSU: 99.8%, *rpb2*: 100%, *tub2*: 99.7%). To the best of our knowledge, this is the first report of *C. pseudoglobosum* associated with *A. sinicus* worldwide.

**Figure 8:**
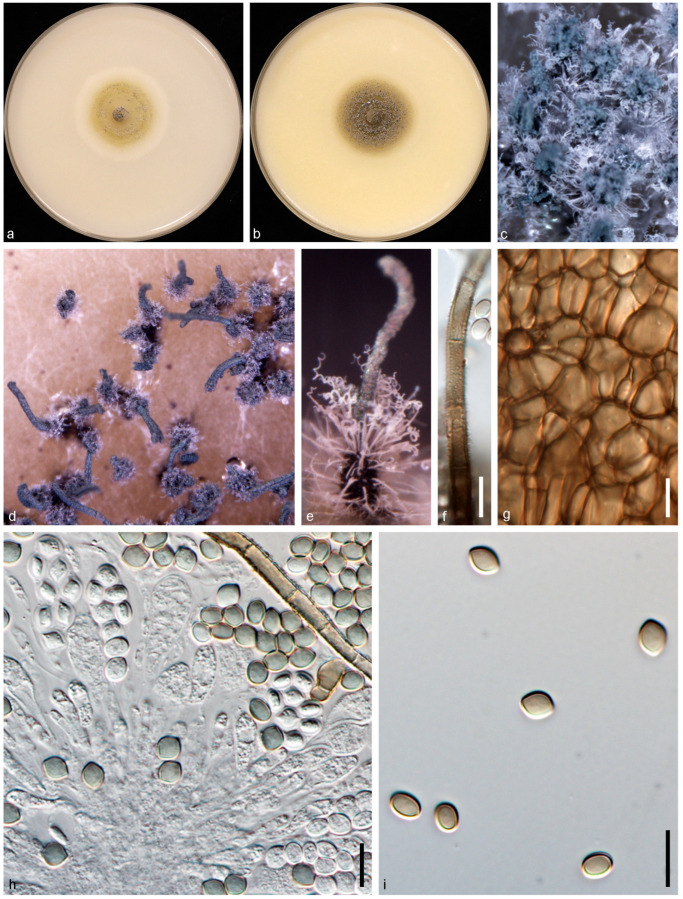
*Collariella pachypodioides* (CGMCC 3.24316/NSYB1). (**a**,**b**) Colonies on OA (**a**) and CMA (**b**); (**c**–**e**) mature ascomata on OA, top view (**c**,**d**) and side view (**e**); (**f**) terminal hairs; (**g**) ascomatal wall; (**h**) asci; (**i**) ascospores. Scale bars: (**f**,**i**) = 10 μm.

*Collariella pachypodioides* (L.M. Ames) X.Wei Wang and Houbraken, Studies in Mycology 101: 167. 2022. (Figure 8).

Description and Illustration—Wang et al. [1].

Material examined: CHINA: Sichuan Province: Guangan, Yuechi, 106°44′07.9″ E, 30°53′91.8″ N, isolated from healthy leaves of *Vicia villosa*, 19 Mar. 2020, Y.H. Wu (dry culture HMAS 352365, living culture CGMCC 3.24316 = NSYB1).

Genbank accession number: ITS: PP062898, LSU: PP062908, *rpb2*: PP067877, *tub2*: PP067867.

Notes: In this study, we obtained an isolate (CGMCC 3.24316) from healthy leaves of *V. villosa* that showed morphological similarity to the *Collariella* species, and based on both morpho-molecular data, it has been identified as *Collariella pachypodioides*. In multi-locus phylogeny, our isolate is in a clade with the ex-type strain of *C. pachypodioides* (CBS 164.52), with a high bootstrap support (MLBS: 100%; MPBS: 100%; BYPP: 1.00). When comparing the nucleotide differences of our isolate with the ex-type strain of *C. pachypodioides* (CBS 164.52), we observe high nucleotide similarity in four loci (ITS: 100%, LSU: 100%, *rpb2*: 97.6%, *tub2*: 98.2%). To the best of our knowledge, this is the first report of *C. pseudoglobosum* associated with *V. villosa* worldwide.

**Figure 9:**
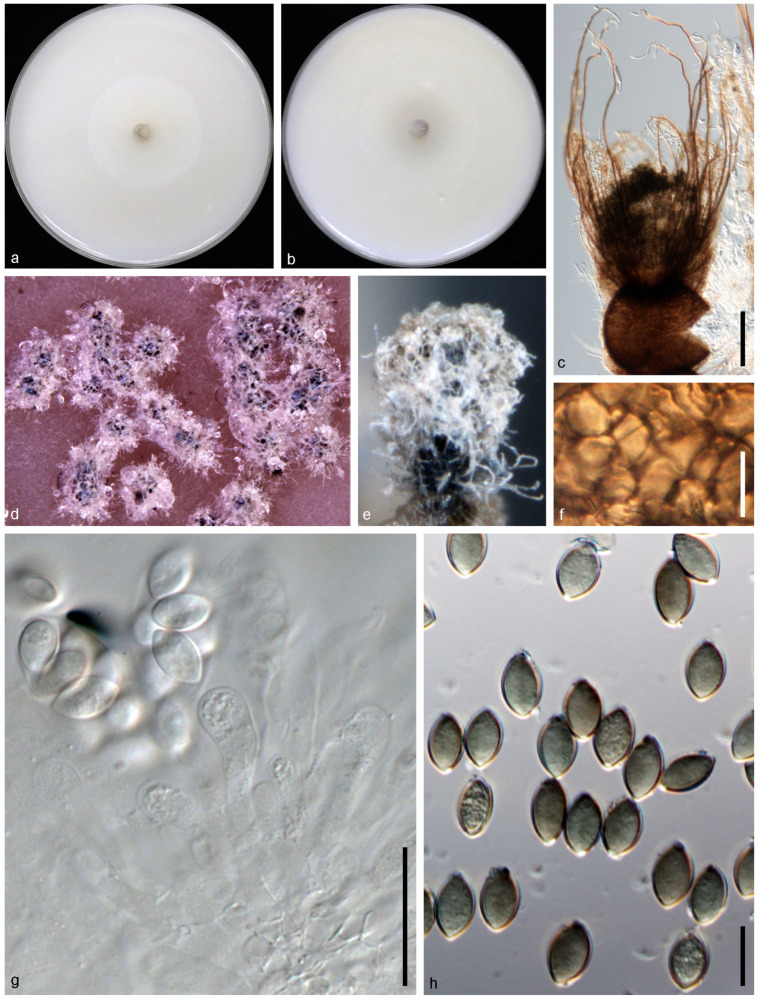
*Subramaniula henanensis* (CGMCC 3.24319/XZYB3d). (**a**,**b**) Colonies on OA (**a**) and CMA (**b**); (**c**) ascomata mounted in lactic acid; (**d**,**e**) mature ascomata on OA, top view (**d**) and side view (**e**); (**f**) ascomatal wall; (**g**) asci; (**h**) ascospores. Scale bars: (**f**,**h**) = 10 μm, (**g**) = 20 μm, (**c**) = 100 μm.

*Subramaniula henanensis* N. Qian and W.S. Zhao, sp. nov. (Figure 9).

MycoBank MB851544.

Etymology—‘henanensis’ refers to the Henan province in China, where the sample was collected.

Diagnosis: *S. henanensis* was distinguished from the phylogenetically most closely related species, *S. cristata* and *S. cuniculorum*, by unique single nucleotide polymorphisms in the four loci used in this study.

Type: CHINA, Henan Province, Xinyang, Gushi, 114°53′14.0″ E, 32°20′27.7″ N, isolated from healthy leaves of *A. sinicus*, 11 Apr. 2021, W. S. Zhao (holotype HMAS 352370, ex-type living culture CGMCC 3.24319 = XZYB3d).

Genbank accession number: ITS: PP062904, LSU: PP062914, *rpb2*: PP067883, *tub2*: PP067873.

Descriptions: Asexual morph: not observed. Sexual morph: Ascomata superficial, pale mouse gray to mouse gray in reflected light owing to ascomatal hairs, ostiolate, subglobose or ovate, 130–280 μm high, 100–240 μm diam. Ascomatal wall brown, textura angularis in surface view. Terminal hairs erect, dark brown, 2.5–3.5 μm diam near the base, apically irregularly-curved and branched repeatedly, fading and tapering towards the tips. Lateral hairs sparse, brown, seta-like, fading and tapering towards the tips. Asci fasciculate, fusiform or clavate, spore-bearing part 28–38 × 11–17 μm, stalks 11–42 μm long, with 8 irregularly arranged ascospores, evanescent. Ascospores olivaceous when mature, citiform and elipsoid, 9.3–11.6 × 5.8–7.3 μm (av. ± SD: 10.6 ± 0.4 × 6.7 ± 0.3 μm), with an apical germ pore.

Cultural characteristics: Colonies on OA reaching 35 mm in diameter after 7 d at 25 °C, circular with a smooth margin, usually without aerial hyphae, without color exudates. Colonies on CMA reaching 40 mm in diameter after 7 d at 25 °C, circular with smooth margin, usually without aerial hyphae, with light gray exudates.

Notes: In this study, we obtained an isolate (CGMCC 3.24319) from healthy leaves of *A. sinicus* and based on multi-loci phylogenetic analysis, this isolate formed a sister clade to two known *Subramaniula* species, *S. cristata* (CBS 156.52T, DTO 324-H7) and *S. cuniculorum* (CBS 800.83R, CBS 121.57), and one new species introduced in this study (*S. sichuanensis* sp. nov) with high statistical support (MLBS: 100%, MPBS: 100%, BYPP: 1.00). Morphologically, *S. henanensis* can be distinguished from *S. cristata* by its smaller ascomata (size of S. cristata: 240–390 μm high, 200–300 μm diam), thinner terminal hairs (size of *S. cristata*: 4.5–5.5 μm diam near the base), wider spore-bearing part (size of *S. cristata*: 9–12 μm), and longer stalks (size of *S. cristata*: 12–30 μm) [37]. No ex-type culture of *S. cuniculorum* is available now, so we cannot compare the morphological differences between them. *S. henanensis* can be distinguished from *S. sichuanensis* by the different terminal hairs (*S. sichuanensis* has two types of terminal hairs), smaller ascomata (size of *S. sichuanensis*: 130–320 μm diam), and smaller spore-bearing part (size of *S. sichuanensis*: 30–46 × 13–21 μm). Based on morphological and phylogenetic data, here we assigned this isolate (CGMCC 3.24319) as a new species, named *S. henanensis* sp. nov.

When comparing the nucleotide differences of our isolate with the type or representative strains of *S. cristata* (CBS 156.52T), *S. cuniculorum* (CBS 800.83R), and *S. sichuanensis* (CGMCC 3.24317T), we observe nucleotide differences in all gene regions as follows: ITS: 0.4%, *rpb2*: 0.2%, and *tub2*: 6.8% (between *S. henanensis* and *S. cristata*); ITS: 0.4%, *rpb2*: 2.1%, and *tub2*: 0.5% (between *S. henanensis* and *S. cuniculorum*); and ITS: 0.2%, LSU: 4.8%, *rpb2*: 7.0%, and *tub2*: 8.1% (between *S. henanensis* and *S. sichuanensis*), respectively. Furthermore, the PHI test (w = 0.7866) did not detect any recombination events between *S. henanensis* and its sister taxon (Figure 3b).

**Figure 10:**
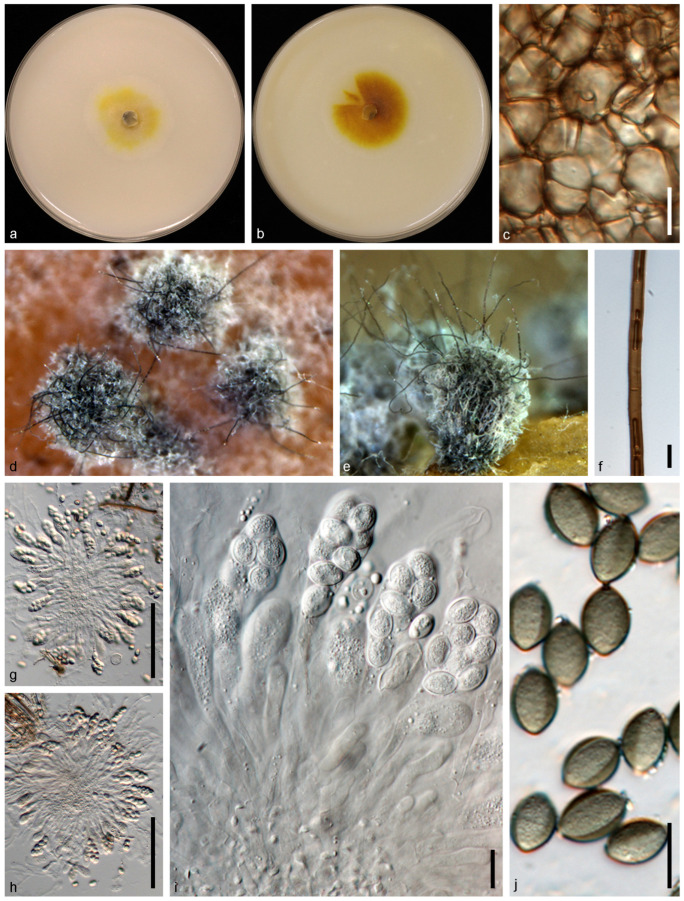
*Subramaniula sichuanensis* (CGMCC 3.24317/NZYA1). (**a**,**b**) Colonies on OA (**a**) and CMA (**b**); (**c**) ascomatal wall; (**d**,**e**) mature ascomata on OA, top view (**d**) and side view (**e**); (**f**) terminal hairs; (**g**–**i**) asci; (**h**) ascospores. Scale bars: (**f**,**h**–**j**) = 10 μm, (**g**,**h**) = 100 μm.

*Subramaniula sichuanensis* N. Qian and W.S. Zhao, sp. nov. (Figure 10).

MycoBank MB851545.

Etymology—‘sichuanensis’ refers to the Sichuan province in China, where the sample was collected.

Diagnosis: *S. sichuanensis* was distinguished from the phylogenetically most closely related species, *S. cristata* and *S. cuniculorum*, by unique single nucleotide polymorphisms in the four loci used in this study.

Type: CHINA: Sichuan Province: Guangan, Yuechi, 106°44′07.9″ E, 30°53′91.8″ N, isolated from the leaf of *A. sinicus*, 19 Mar. 2020, Y.H. Wu (holotype HMAS 352368, ex-type culture CGMCC 3.24317 = NZYA1.

Genbank accession number: ITS: PP062895, LSU: PP062905, *rpb2*: PP067874, *tub2*: PP067864.

Descriptions: Asexual morph not observed. Sexual morph: Ascomata superficial, mouse gray to grayish-gray in reflected light owing to ascomatal hairs, ostiolate, subglobose or ovate, 110–290 μm high, 130–320 μm diam. Ascomatal wall brown, textura angularis in surface view. Terminal hairs of two types: type I numerous, shorter, often erect in the lower part, irregularly arcuate to loosely and irregularly coiled in the upper part, brown, septate, tapering and fading towards the tips; type II only a few, longer, erect or flexuous, brown, septate, tapering towards the tips. Lateral hairs sparse, brown, seta-like, fading and tapering towards the tips. Asci fasciculate, fusiform or clavate, spore-bearing part 30–46 × 13–21 μm, stalks 16–41 μm long, with 8 irregularly arranged ascospores, evanescent. Ascospores olivaceous when mature, fusiform, 9.5–11.9 × 6.3–7.9 μm (av. ± SD: 10.8 ± 0.6 × 7.1 ± 0.4 μm), with an apical germ pore.

Cultural characteristics: Colonies on OA reaching 30 mm in diameter after 7 d at 25 °C, circular, usually without aerial hyphae, with luteous exudates. Colonies on CMA reaching 35 mm in diameter after 7 d at 25 °C, circular, usually without aerial hyphae, with ochreous to orange exudates.

Additional material examined: CHINA: Sichuan Province: Guangan, Yuechi, 106°44′07.9″ E, 30°53′91.8″ N, isolated from the leaf of *A. sinicus*, 19 Mar. 2020, Y.H. Wu (dry cculture HMAS 352369, culture CGMCC 3.24318 = NZYA2).

Genbank accession number: ITS: PP062896, LSU: PP062906, *rpb2*: PP067875, *tub2*: PP067865.

Notes: In this study, we obtained two isolates (CGMCC 3.24317, CGMCC 3.24318) from healthy leaves of *A. sinicus* and based on multi-loci phylogenetic analysis, this isolate formed a sister clade to two known *Subramaniula* species, *S. cristata* (CBS 156.52T, DTO 324-H7) and *S. cuniculorum* (CBS 800.83R, CBS 121.57), and one new species introduced in this study (*S. henanensis* sp. nov) with high statistical support (MLBS: 100%, MPBS: 100%, BYPP: 1.00). Morphologically, *S. sichuanensis* can be distinguished from *S. cristata* by its smaller ascomata (size of *S. cristata*: 240–390 μm high, 200–300 μm diam), different terminal hairs (*S. cristata* just has one type), bigger spore-bearing part (size of *S. cristata* size: 23–40 × 9–12 μm), and longer stalks (size of *S. cristata*: 12–30 μm) [37]. No ex-type culture of *S. cuniculorum* is available now, so we cannot compare the morphological differences between them. The morphological difference between *S. henanensis* and *S. sichuanensis* refer to the previous note. Based on morphological and phylogenetic data, here we assigned these isolates (CGMCC 3.24317, CGMCC 3.24318) as a new species, named *S. sichuanensis* sp. nov.

When comparing the nucleotide differences of our isolate with the type or representative strains of *S. cristata* (CBS 156.52T) and *S. cuniculorum* (CBS 800.83R), we observe nucleotide differences in all gene regions as follows: ITS: 0.2%, LSU: 0.4%, *rpb2*: 8.2%, and *tub2*: 8.2% (between *S. sichuanensis* and *S. cristata*); and ITS: 0.2%, LSU: 0.4%, *rpb2*: 8.6%, and *tub2*: 7.7% (between *S. sichuanensis* and *S. cuniculorum*), respectively. Furthermore, the PHI test (w = 0.7866) did not detect any recombination events between *S. henanensis* and its sister taxon (Figure 3b).

### 3.4. Result of Dual Culture Test

Dual culture experiments showed that 10 isolates of Chaetomiaceae exhibited varying degrees against the tested phytopathogens (Figures S1–S15). The relative inhibiting effect indexes were caculated and are shown in Table 3, in which the higher the number, the more intense the effect. Notably, the C. coarctatum isolate NSJA2 showed significantly (*p* < 0.05) higher relative inhibiting effects against 14 of the 15 phytopathogens (Table 3). Meanwhile, the relative inhibiting effects of NSJA2 against four pathogens, namely *S. astragali* (XZYB6f), *F. graminearum* (F0609), *F. oxysporum* (FOC), and *S. minor* (HJ5), were even more than 10, indicating higher inhibiting effects. The radial inhibited rate of the phytopathogens ranged from 54% to 81% (Table S2), while the radial inhibited rate of NSJA2 by individual phytopathogens ranged from 4% to 30% (Table S3). Moreover, the radial inhibited rates of NSJA2 by eight tested phytopathogens were significantly lower than those of the other nine isolates (Table S3), while the radial inhibiting proportion of NSJA2 against six tested phytopathogens was significantly higher than those of the other nine isolates (Table S2). These results indicate that the isolate NSJA2 has excellent potential as a biocontrol resource.

### 3.5. Salt Tolerance Ability of NSJA2

**Figure 11:**
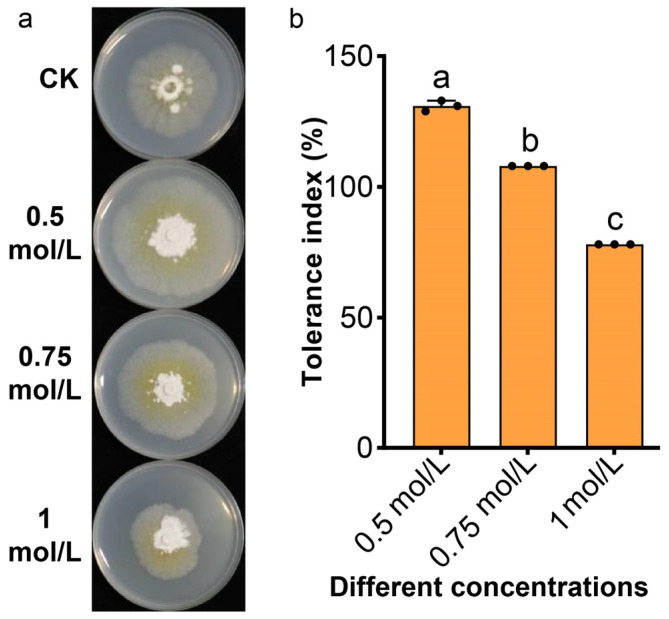
Tolerance of NSJA2 to different concentrations of NaCl in PDA culture medium. (**a**) Effect of NaCl on the mycelial growth of NSJA2 in PDA culture medium; (**b**) tolerance index of NSJA2 to different concentrations of NaCl in PDA culture medium. Data are the means of three samples. Vertical bars represent the standard error of means (SEM). Lowercase letters represent significant differences between treatments (*p* < 0.05).

To investigate the salt tolerance of NSJA2, the fungus was cultured on PDA plates with varying concentrations (0.5, 0.75, and 1 mol/L of NaCl). When the salt concentration in the culture medium is 0.5 mol/L or 0.75 mol/L, the tolerance index of NSJA2 is more than 100% (Figure 11), suggesting that a lower concentration of NaCl can promote the growth of NSJA2. Even when the salt concentration was raised to 1 mol/L, the tolerance index still reached 77.55% (Figure 11). These results indicate that NSJA2 possesses excellent salt tolerance.

## 4. Discussion

In China, *Astragalus sinicus* and *Vicia villosa* are commonly used as green manure and cover crops. In the previous study, we described seven new fungal species and twenty-four new host associations of different fungal species on *A. sinicus* and *V. villosa*, using a culture-dependent method. Mycobiome analysis allowed to obtain a total of 178 Operational Taxonomic Units (OTUs), which were further assigned to 21 classes, 48 orders, 66 families, and 74 genera, indicating that these crops carry a significant number of unexplored fungal communities. Notably, several species with the potential for the prevention of plant diseases were found in the identified OTUs [26].

In present study, we have identified several species that belong to the family *Chaetomiaceae*, from healthy plant parts of *A. sinicus* and *V. villosa*. In total, we have obtained ten fungal strains, belonging to five fungal genera. Later, based on morpho-molecular phylogenetic analysis, these isolates were identified as *Achaetomium astragali*, *Botryotrichum murorum*, *Chaetomium coarctatum*, *C. pseudocochliodes*, *C. pseudoglobosum*, *Collariella pachypodioides*, *S. henanensis*, and *S. sichuanensis*. The *Chaetomium coarctatum* (NSJA2) was isolated from the stem of *V. villosa*, while the other species were isolated from the leaf of *V. villosa* or *Astragalus sinicus*. Among the identified species, *A. astragali*, *S. henanensis*, and *S. sichuanensis* were described as new species, while the other five species were reported for the first time on these host plants. This may indicate the abundant fungal species associated with these cover crops, and therefore further studies are recommended to evaluate the total fungal diversity of these green manure crops.

In this study, we have obtained a fungal species (CGMCC 3.24315) that is phylogenetically classified into the *Achaetomium* genus. However, we were unable to compare the morphological characters with other known *Achaetomium* species, because our isolate does not produce the sexual morph characters on the culture. All other previously reported *Achaetomium* species are only known from their sexual morph characters [44,45,46,47].

Some species of *Chaetomiaceae* have been reported to have potential biocontrol activity. One such species is *C. globosum*, which produces 300 active secondary metabolites and has been used as a biological control agent to manage *Sclerotinia* disease in rape and tan spot disease in wheat [6,7], while *C. coarctatum* has been shown to inhibit the growth of *F. avenaceum*, which causes root rot on *Coptis chinensis* [23]. In the present study, we showed that all 10 isolates representing five genera of *Chaetomiaceae* from the two cover crops exhibited antagonistic effects on different phytopathogens. Among them, the *C. coarctatum* isolate NSJA2 showed a higher relative inhibition effect on 14 out of the 15 phytopathogens tested in this study, with an inhibition rate of over 50%. Moreover, the relative inhibition effect indexes of NSJA2 against four phytopathogens, including *F. graminearum*, *F. oxysporum* f. sp. *cucumerinum*, *S. astragali*, and *S. minor*, were over 10. A previous study reported that *C. pseudocochliodes* causes petal blight disease on *Camellia reticulata* in China [48]. However, our results showed that the *C. pseudocochliodes* isolate NSYB2 demonstrated a strong inhibitory effect on phytopathogens, such as *A. alternata* and *B. cinerea*. These findings underscore the potential of the *Chaetomiaceae* species from cover crops to be a valuable resource for developing biocontrol agents. In particular, the *C. coarctatum* isolate NSJA2 shows promise as a potential biocontrol agent. These findings indicate that the species of *Chaetomiaceae* from cover crops can provide abundant fungal resources, and *C. coarctatum* NSJA2 is a potential biocontrol agent.

Recent studies have shown that certain endophytic fungi, including *Trichoderma longibrachiatum* and *C. coarctatum*, have the ability to tolerate high levels of salt. This attribute makes them useful in preventing fungal diseases and promoting plant growth in saline conditions [49,50]. Besides the antagonistic effect on various phytopathogens, NSJA2 exhibited remarkable salt tolerance in saline medium, with a tolerance index greater than 100% when the salt concentration in the medium was below 0.5 mol/L. Even if the salt concentration in the culture medium reached 1 mol/L, the tolerance index of NSJA2 still reached 77.55% (Figure 11). This is significant because a NaCl solution exceeding 200 mM can cause rice death [51]. Our results suggest that the *C. coarctatum* isolate NSJA2 has good adaptability to high salt environments and can be used for the prevention of plant disease even in saline conditions.

## 5. Conclusions

We identified eight entophytic fungi species of five genera in *Chaetomiaceae* from the ten fungal isolates collected from the two green manure and cover crops (*A. sinicus* and *V. villosa*). Among these, we found three were new species and five host records, further indicating the taxonomic richness of both plant species. The isolates of all eight *Chaetomiaceae* species exhibited antagonistic effects on multiple phytopathogens. Notably, the *C. coarctatum* isolate NSJA2 exhibited significant relative inhibition effects on 14 out of the 15 phytopathogens tested in this study, indicating its broad-spectrum antagonistic effects. Additionally, NSJA2 exhibited excellent salt tolerance. Together, our results offer several potential fungal biocontrol resources, with NSJA2 being particularly promising for further exploitation and utilization as a novel biocontrol agent.

## Figures and Tables

**Table 1:** Fungal isolates obtained in this study.

Isolate Number	Host	Substrate	Locality	NCBI-BLAST Result
g6	*Astragalus sinicus*	leaf	Henan province, Xinyang city	*Achaetomium* sp.
NSYB3	*Vicia villosa*	leaf	Sichuan province, Guangan city	*Botryotrichum* sp.
NSYB4	*Vicia villosa*	leaf	Sichuan province, Guangan city	*Botryotrichum* sp.
NSYB1	*Vicia villosa*	leaf	Sichuan province, Guangan city	*Collariella* sp.
NSJA2	*Vicia villosa*	stem	Sichuan province, Guangan city	*Chaetomium* sp.
NSYB2	*Vicia villosa*	leaf	Sichuan province, Guangan city	*Chaetomium* sp.
GXZYB1	*Astragalus sinicus*	leaf	Guangxi province, Nanning city	*Chaetomium* sp.
NZYA1	*Astragalus sinicus*	leaf	Sichuan province, Guangan city	*Subramaniula* sp.
NZYA2	*Astragalus sinicus*	leaf	Sichuan province, Guangan city	*Subramaniula* sp.
XZYB3d	*Astragalus sinicus*	leaf	Henan province, Xinyang city	*Subramaniula* sp.

**Table 2:** Model detection results for Bayesian inference.

Dataset	Subst. Model ^a^	NST ^b^	Rate Matrix						*p*-Inv	Gamma	Rates
			[AC]	[AG]	[AT]	[CG]	[CT]	[GT]			
ITS	TIM2 + I + G	6	1.8695	1.5540	1.8695	1.0000	4.9116	1.0000	0.5550	0.9100	gamma
LSU	TIM3ef + I + G	6	0.5820	1.0173	1.0000	0.5820	4.9349	1.0000	0.7490	0.5600	gamma
*rpb2*	TrN + I + G	6	1.0000	4.8611	1.0000	1.0000	8.3943	1.0000	0.4850	1.0410	gamma
*tub2*	HKY + I + G	2	-	-	-	-	-	-	0.3220	2.1740	gamma

^a^ Subst. model = best-fit substitution model. ^b^ NST = number of substitution rate categories.

**Table 3:** Relative inhibiting effects against the tested phytopathogens.

Phytopathogens	10 Isolates of *Chaetomiaceae*				
	g6	GXZYB1	NSYB2	NSJA2	NSYB1
*Fusarium graminearum* F0609	0.42 ± 0.01 ef ^a^	1.19 ± 0.06 cd	0.44 ± 0.09 ef	11.40 ± 1.18 a	1.16 ± 0.15 cde
*Botrytis cinerea* B79	1.86 ± 0.24 c	2.30 ± 0.07 b	1.94 ± 0.08 c	5.72 ± 0.13 a	1.31 ef
*Colletotrichum siamense* CCT1	0.97 ± 0.02 de	2.04 ± 0.01 a	1.43 ± 0.05 b	1.83 ± 0.16 a	1.82 ± 0.11 a
*F. oxysporum* f. sp. *cucumerinum* Foc	0.66 e	1.30 ± 0.06 b	1.04 ± 0.08 c	13.06 a	0.74 ± 0.05 de
*Stemphylium astragali* XZYB6f	2.44 ± 0.18 cd	3.02 ± 0.37 bc	3.02 ± 0.09 bc	10.71 ± 2.71 a	4.25 b
*Sclerotinia minor* HJ5	2.93 ± 0.06 b	1.59 ± 0.36 bc	1.31 ± 0.13 c	12.60 ± 2.76 a	0.38 ± 0.22 c
*S. sclerotiorum* ZJ25	5.47 ± 0.16 b	1.77 ± 0.51 e	2.54 ± 0.11 d	9.78 ± 0.17 a	3.71 ± 0.15 c
*Pyricularia oryzae* P131	0.98 ± 0.09 cd	0.48 ± 0.08 ef	0.32 ± 0.07 f	3.09 ± 0.45 a	1.41 ± 0.11 b
*Botryosphaeria dothidea* B-8-1	0.98 ± 0.03 e	2.83 ± 0.43 c	2.53 ± 0.58 c	7.05 ± 0.28 a	3.47 ± 0.36 b
*Phytophthora capsici* LT263	3.07 ± 0.08 b	2.61 ± 0.11 b	1.36 ± 0.15 c	4.55 ± 0.58 a	2.89 ± 0.60 b
*Verticillium dahliae* V991	1.11 ± 0.12 bc	0.57 ± 0.07 c	0.65 ± 0.04 c	5.40 ± 1.01 a	1.45 ± 0.03 b
*Alternaria brassicae* HEYA2	1.29 ± 0.16 b	1.43 ± 0.47 b	0.60 ± 0.10 c	5.06 ± 0.34 a	0.34 ± 0.08 c
*Alternaria alternate* A33	1.34 ± 0.08 b	1.05 ± 0.03 b	1.09 ± 0.03 b	7.07 ± 1.75 a	1.02 ± 0.16 b
*Boeremia linicola*Y3-3	0.13 e	2.08 ± 0.84 b	0.25 ± 0.02 e	9.13 ± 0.39 a	1.52 ± 0.22 c
*Lasiodiplodia theobromae* CSS-01S	1.42 ± 0.14 d	1.09 ± 0.12 d	2.18 ± 0.15 c	3.88 ± 0.89 a	1.00 ± 0.25 d
	**NSYB3**	**NSYB4**	**NZYA1**	**NZYA2**	**XZYB3d**
*Fusarium graminearum* F0609	2.78 ± 0.14 b	1.58 ± 0.12 c	0.09 ± 0.08 f	0.55 ± 0.32 def	0.65 ± 0.10 def
*Botrytis cinerea* B79	1.06 ± 0.18 f	1.81 ± 0.28 c	1.71 ± 0.14 cd	0.74 ± 0.13 g	1.51 ± 0.14 de
*Colletotrichum siamense* CCT1	1.04 ± 0.09 cd	0.72 ± 0.21 e	1.34 ± 0.09 b	1.39 ± 0.36 b	1.29 ± 0.09 bc
*F. oxysporum* f. sp. *cucumerinum* Foc	1.16 ± 0.14 bc	0.22 f	0.86 ± 0.19 d	0.57 e	1.28 ± 0.17 b
*Stemphylium astragali* XZYB6f	1.74 cde	0.93 ± 0.44 de	1.18 ± 0.19 de	0.72 ± 0.03 e	1.93 ± 0.26 cde
*Sclerotinia minor* HJ5	0.32 ± 0.26 c	0.64 ± 0.09 c	0.89 ± 0.23 c	0.25 ± 0.03 c	0.72 ± 0.20 c
*S. sclerotiorum* ZJ25	0.31 g	0.41 ± 0.05 g	1.16 ± 0.24 f	0.44 ± 0.06 g	1.00 ± 0.23 f
*Pyricularia oryzae* P131	1.14 ± 0.19 bc	0.86 ± 0.19 cde	1.44 ± 0.33 b	0.59 ± 0.30 def	1.10 ± 0.02 bc
*Botryosphaeria dothidea* B-8-1	2.65 ± 0.33 c	1.12 e	0.13 f	0.29 ± 0.03 f	1.77 ± 0.24 d
*Phytophthora capsici* LT263	1.58 ± 0.11 c	0.44 ± 0.02 d	2.57 ± 0.91 b	0.22 d	1.85 ± 0.30 c
*Verticillium dahliae* V991	1.53 ± 0.73 b	0.96 ± 0.17 bc	0.93 ± 0.16 bc	0.85 ± 0.18 bc	0.76 ± 0.07 bc
*Alternaria brassicae* HEYA2	0.32 ± 0.04 c	1.16 ± 0.23 b	0.36 ± 0.13 c	0.41 ± 0.10 c	0.40 ± 0.15 c
*Alternaria alternate* A33	0.51 ± 0.09 b	1.50 ± 0.35 b	0.96 ± 0.17 b	1.29 ± 0.23 b	0.77 ± 0.02 b
*Boeremia linicola* Y3-3	1.15 ± 0.18 cd	0.18 ± 0.07 e	0.58 ± 0.10 de	0.30 ± 0.05 e	0.63 ± 0.29 de
*Lasiodiplodia theobromae* CSS-01S	3.00 ± 01.6 b	1.21 ± 0.44 d	0.97 ± 0.99 d	0.34 ± 0.12 e	0.28 ± 0.05 e

^a^ Lowercase letters represent significant differences between treatments (*p* < 0.05).

## Data Availability

All sequence data generated for this study can be accessed via GenBank: https://www.ncbi.nlm.nih.gov/genbank/, accessed on 14 September 2023. Alignments are available at TreeBase (http://www.treebase.org, accessed on 22 September 2024).

## Supplementary Materials

The following supporting information can be downloaded at: https://www.mdpi.com/article/10.3390/jof10110776/s1, Figure S1. Ten *Chaetomiaceae* isolates dual culture with *Alternaria alternata* A33. Figure S2. Ten *Chaetomiaceae* isolates dual culture with *Alternaria brassicae* HEYA2. Figure S3. Ten *Chaetomiaceae* isolates dual culture with *Boeremia linicola* Y3-3. Figure S4. Ten *Chaetomiaceae* isolates dual culture with *Botryosphaeria dothidea* B-8-1. Figure S5. Ten *Chaetomiaceae* isolates dual culture with *Botrytis cinerea* B79. Figure S6. Ten *Chaetomiaceae* isolates dual culture with *Colletotrichum siamense* CCT1. Figure S7. Ten *Chaetomiaceae* isolates dual culture with *Fusarium graminearum* F0609. Figure S8. Ten *Chaetomiaceae* isolates dual culture with *Fusarium oxysporum* f. sp. *cucumerinum* Foc. Figure S9. Ten *Chaetomiaceae* isolates dual culture with *Lasiodiplodia theobromae* CSS-01S. Figure S10. Ten *Chaetomiaceae* isolates dual culture with *Sclerotinia minor* HJ5. Figure S11. Ten *Chaetomiaceae* isolates dual culture with *Sclerotinia sclerotiorum* ZJ25. Figure S12. Ten *Chaetomiaceae* isolates dual culture with *Stemphylium astragali* XZYB6f. Figure S13. Ten *Chaetomiaceae* isolates dual culture with *Phytophthora capsici* LT263. Figure S14. Ten *Chaetomiaceae* isolates dual culture with *Pyricularia oryzae* P131. Figure S15. Ten *Chaetomiaceae* isolates dual culture with *Verticillium dahlia* V991. Table S1. Details of the reference sequence in this study. Table S2. Radial inhibited rate (%) of 10 isolates of *Chaetomiaceae* in dual culture. Table S3. Radial inhibited rate (%) of phytopathogens in dual culture.
