# Supplementary material for: Three New Species and Five New Host Records from Chaetomiaceae with Anti-Phytopathogenic Potential from Cover Crops Astragalus sinicus and Vicia villosa
Source: J Fungi (Basel). 2024 Nov 8;10(11):776. doi: 10.3390/jof10110776 (PMC11595803; doi:10.3390/jof10110776)
Supplement: Supplementary file 1 [file jof-10-00776-s001.zip › jof-3277676-supplementary.pdf]

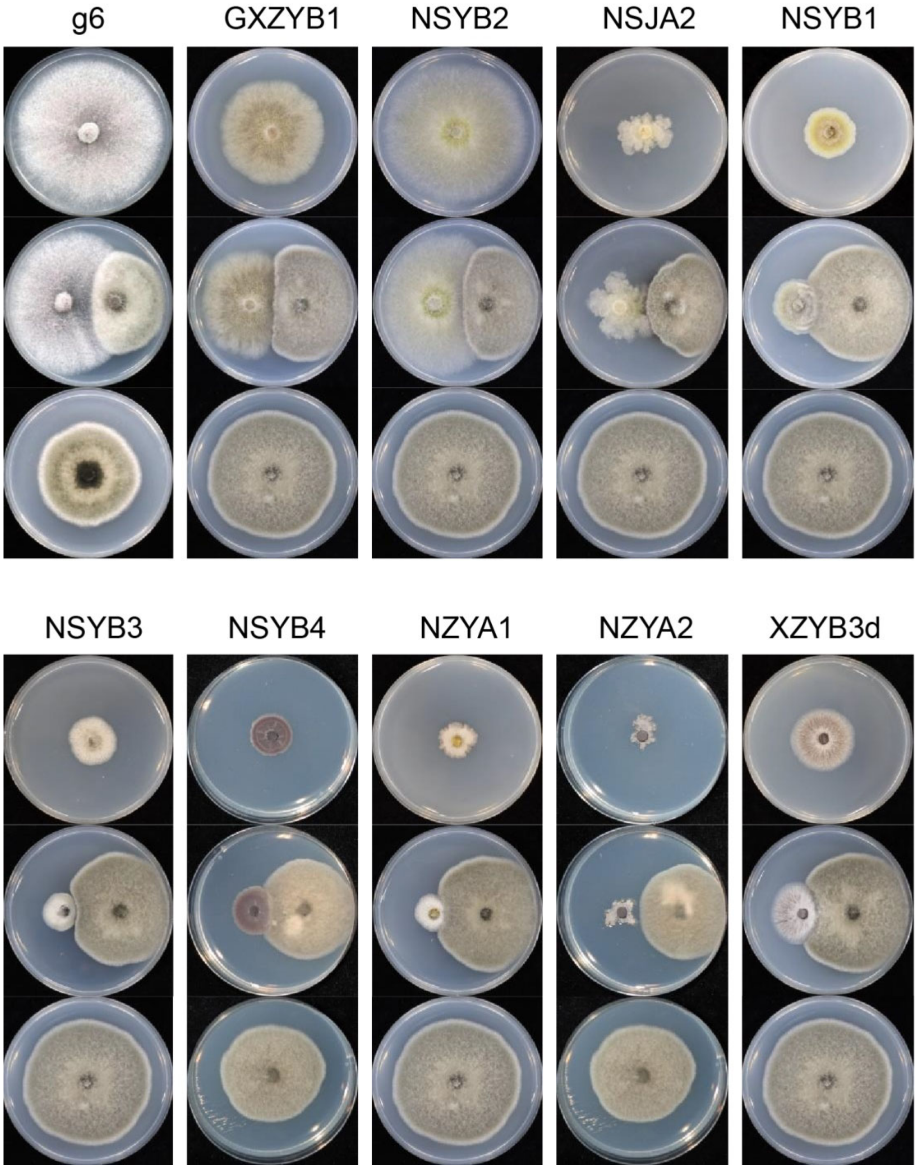

Fig. S1. Ten *Chaetomiaceae* isolates dual culture with *Alternaria alternata* A33. The top, middle, and bottom three colonies in each part of the figure are: *Chaetomiaceae* isolates cultured separately, *Chaetomiaceae* isolates with A33 dual culture, and A33 cultured separately.

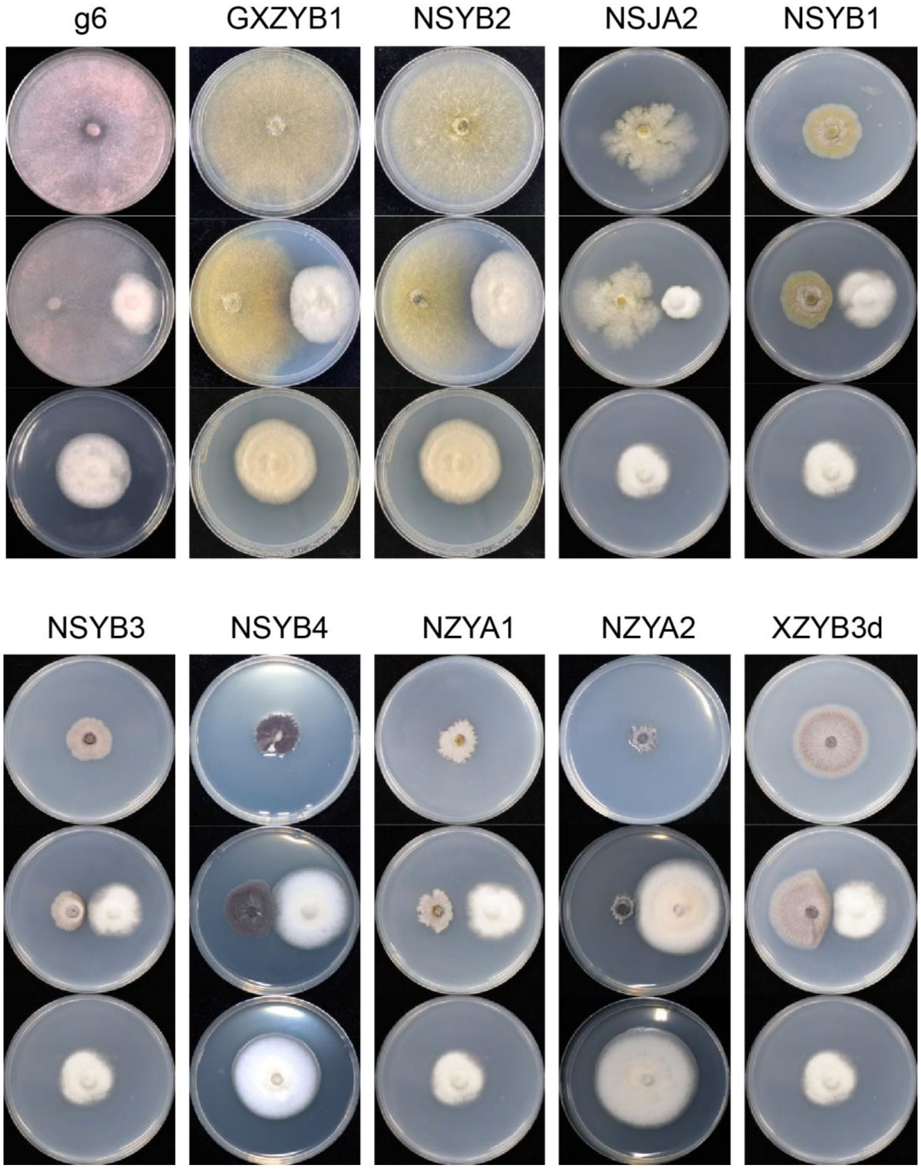

Fig. S2. Ten *Chaetomiaceae* isolates dual culture with *Alternaria brassicae* HEYA2. The top, middle, and bottom three colonies in each part of the figure are: *Chaetomiaceae* isolates cultured separately, *Chaetomiaceae* isolates with HEYA2 dual culture, and HEYA2 cultured separately.

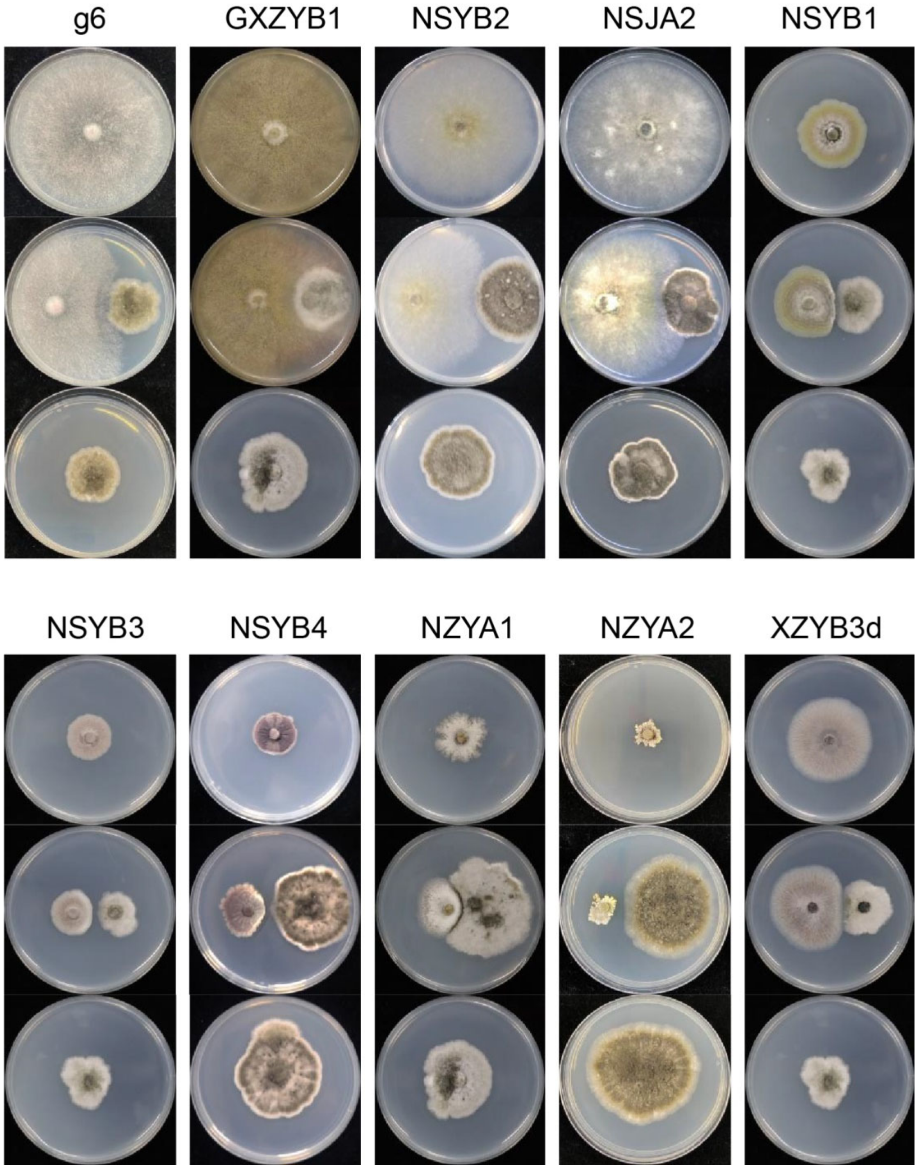

Fig. S3. Ten *Chaetomiaceae* isolates dual culture with *Boeremia linicola* Y3-3. The top, middle, and bottom three colonies in each part of the figure are: *Chaetomiaceae* isolates cultured separately, *Chaetomiaceae* isolates with Y3-3 dual culture, and Y3-3 cultured separately.

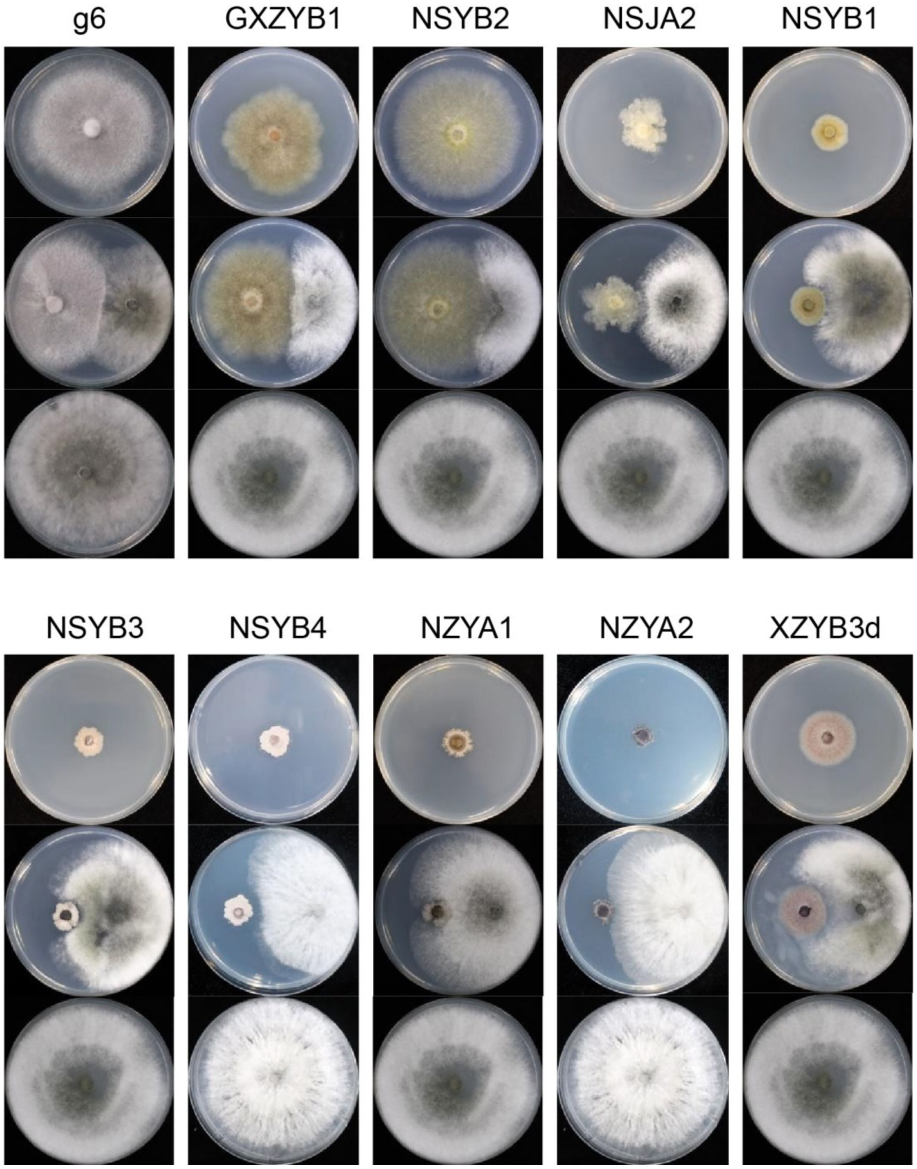

Fig. S4. Ten *Chaetomiaceae* isolates *dual culture* with *Botryosphaeria dothidea* B-8-1. The top, middle, and bottom three colonies in each part of the figure are: *Chaetomiaceae* isolates cultured separately, *Chaetomiaceae* isolates with B-8-1 dual culture, and B-8-1 cultured separately.

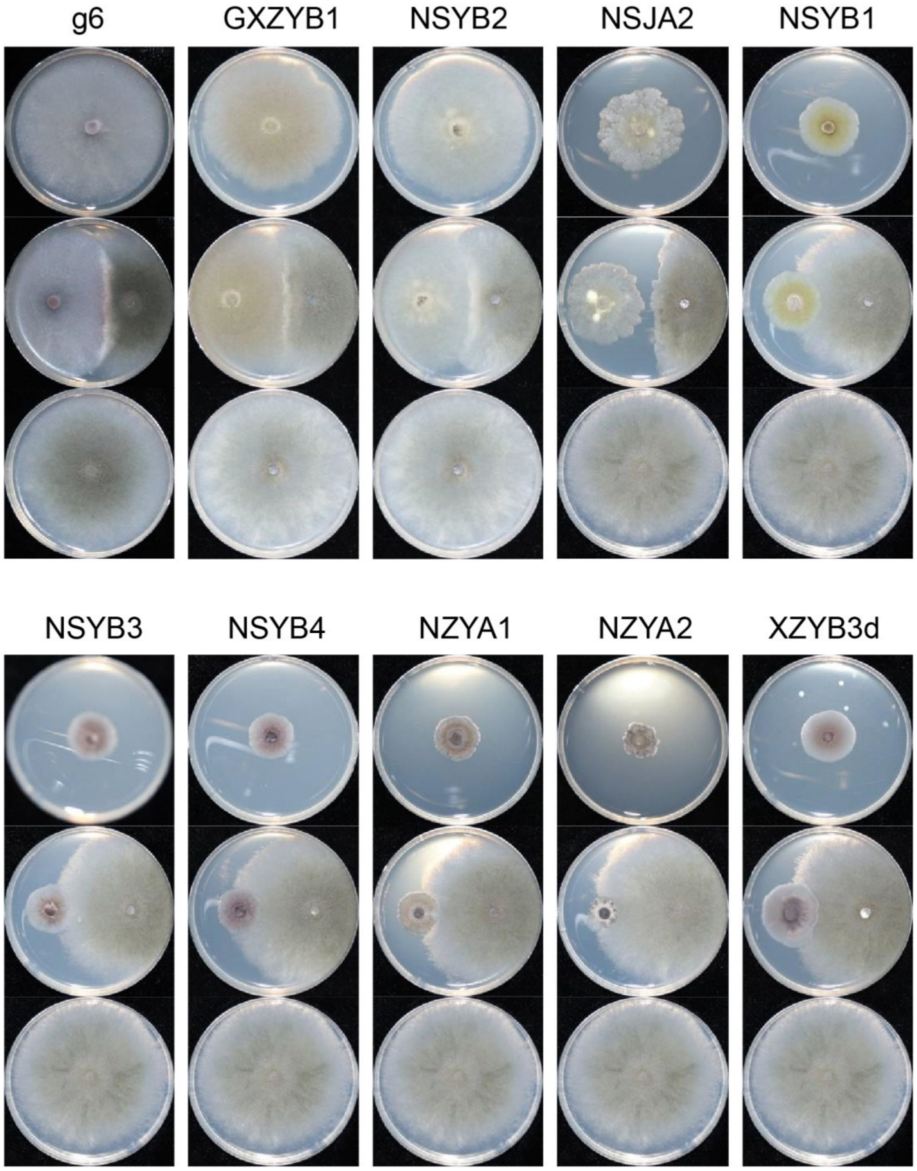

Fig. S5. Ten *Chaetomiaceae* isolates dual culture with *Botrytis cinerea* B79. The top, middle, and bottom three colonies in each part of the figure are: *Chaetomiaceae* isolates cultured separately, *Chaetomiaceae* isolates with B79 dual culture, and B79 cultured separately.

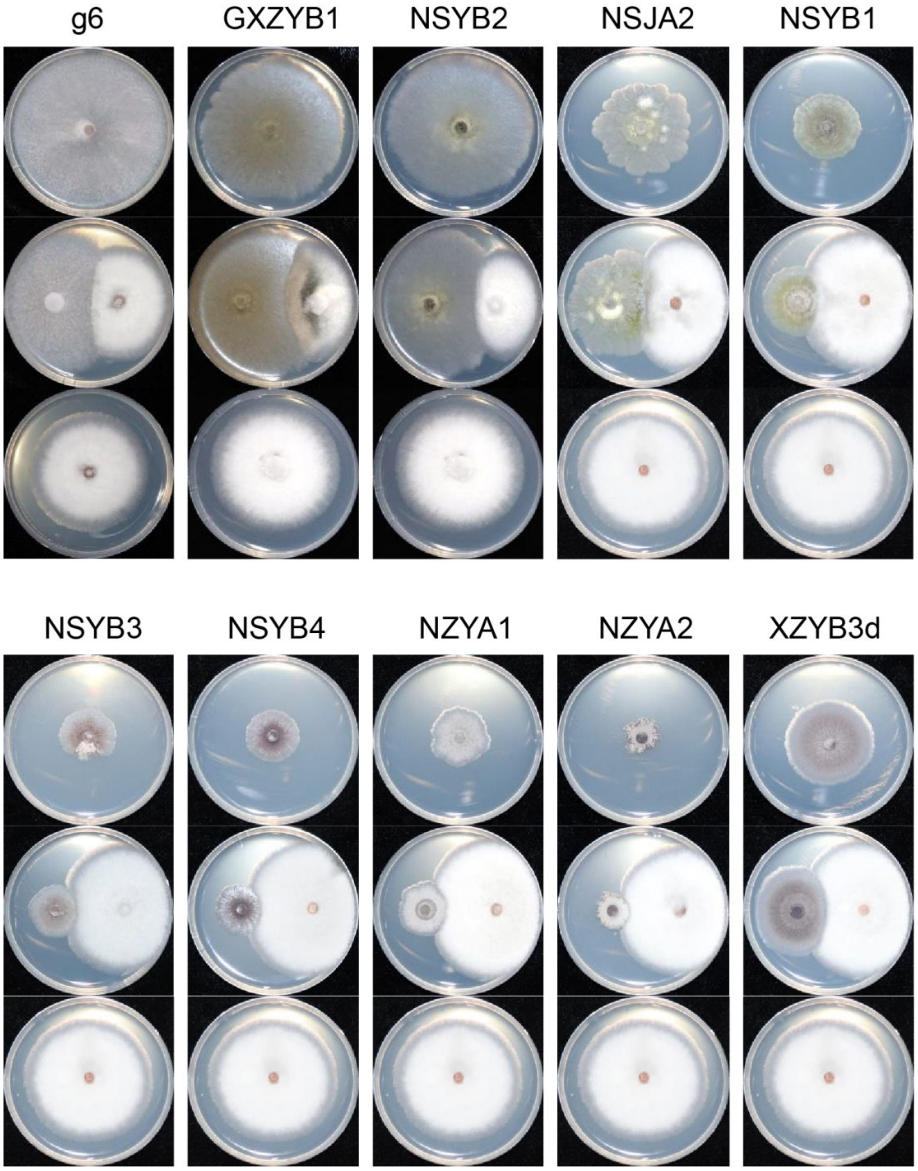

Fig. S6. Ten *Chaetomiaceae* isolates dual culture with *Colletotrichum siamense* CCT1. The top, middle, and bottom three colonies in each part of the figure are: *Chaetomiaceae* isolates cultured separately, *Chaetomiaceae* isolates with CCT1 dual culture, and CCT1 cultured separately.

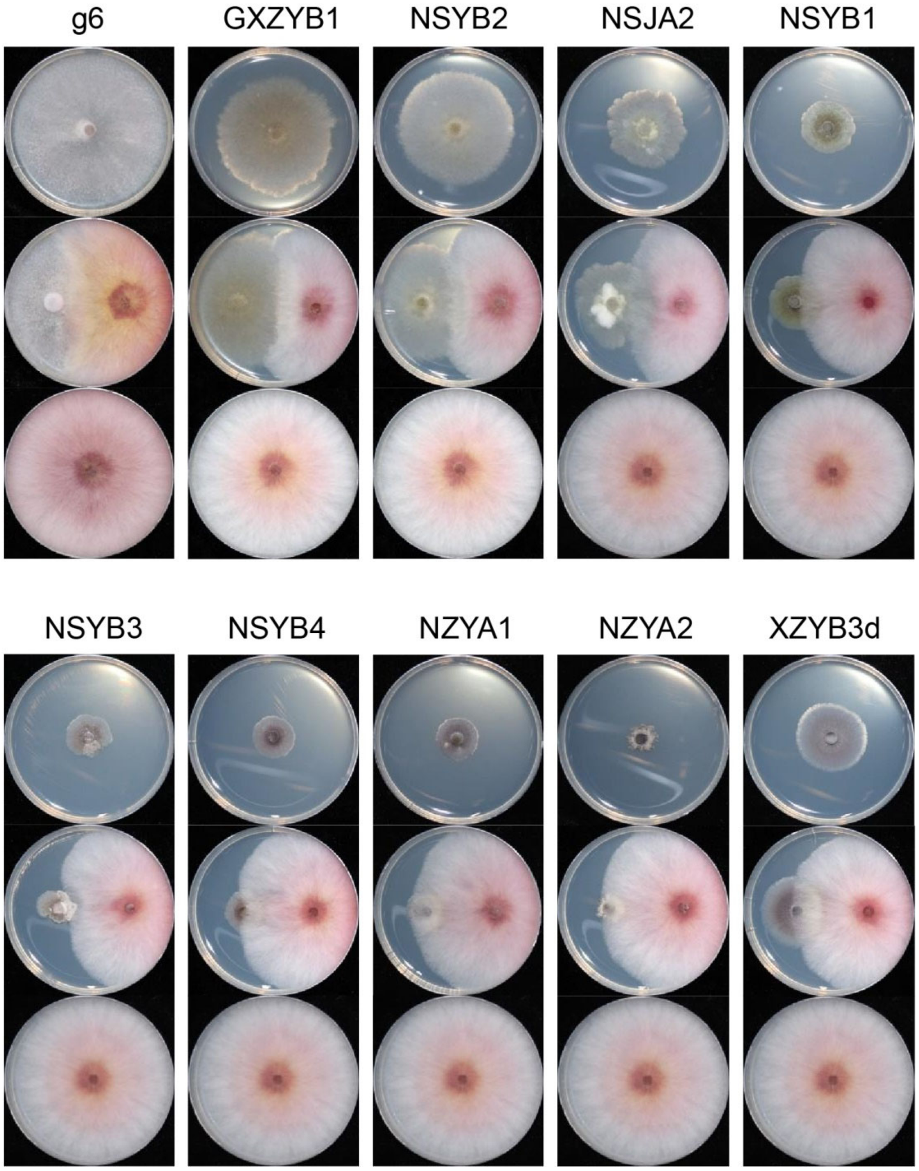

Fig. S7. Ten *Chaetomiaceae* isolates dual culture with *Fusarium graminearum* F0609. The top, middle, and bottom three colonies in each part of the figure are: *Chaetomiaceae* isolates cultured separately, *Chaetomiaceae* isolates with F0609 dual culture, and F0609 cultured separately.

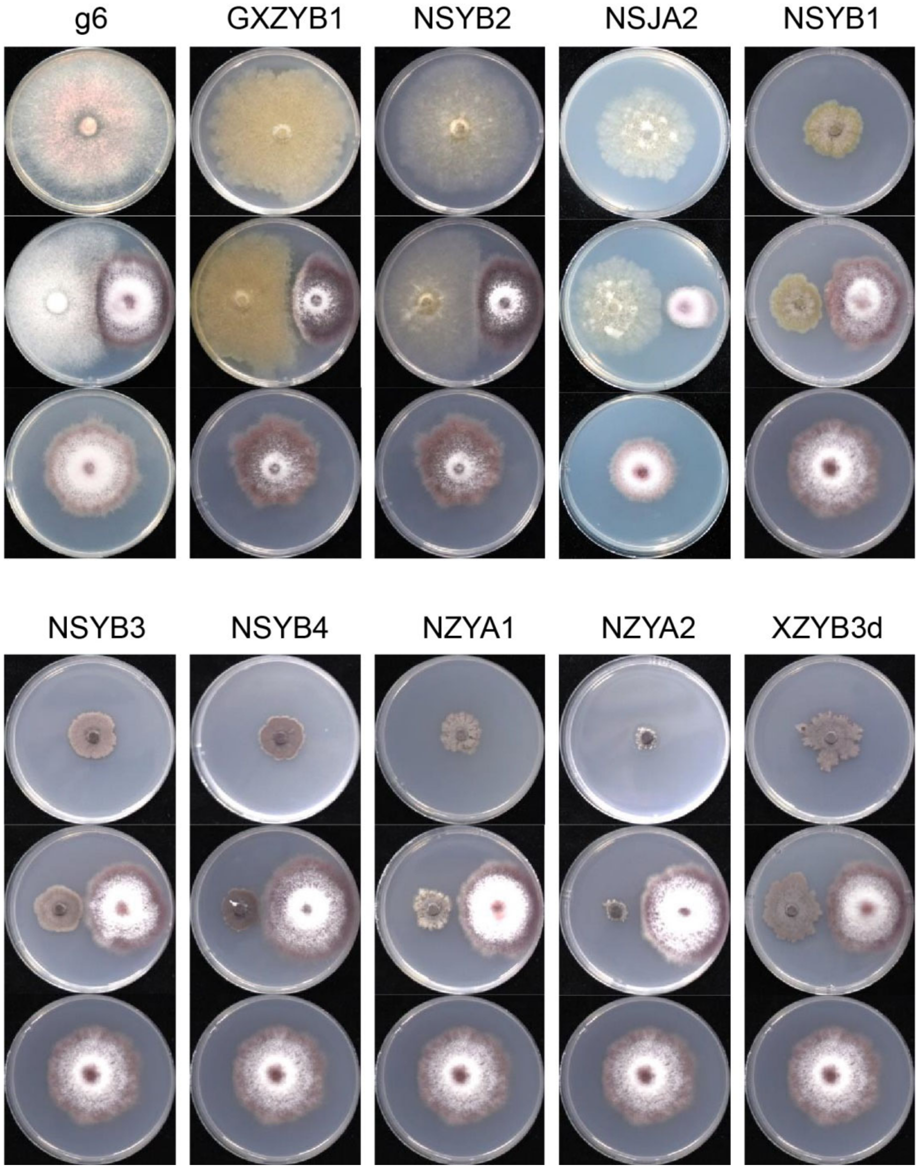

Fig. S8. Ten *Chaetomiaceae* isolates dual culture with *Fusarium oxysporum f. sp. cucumerinum* Foc. The top, middle, and bottom three colonies in each part of the figure are: *Chaetomiaceae* isolates cultured separately, *Chaetomiaceae* isolates with Foc dual culture, and Foc cultured separately.

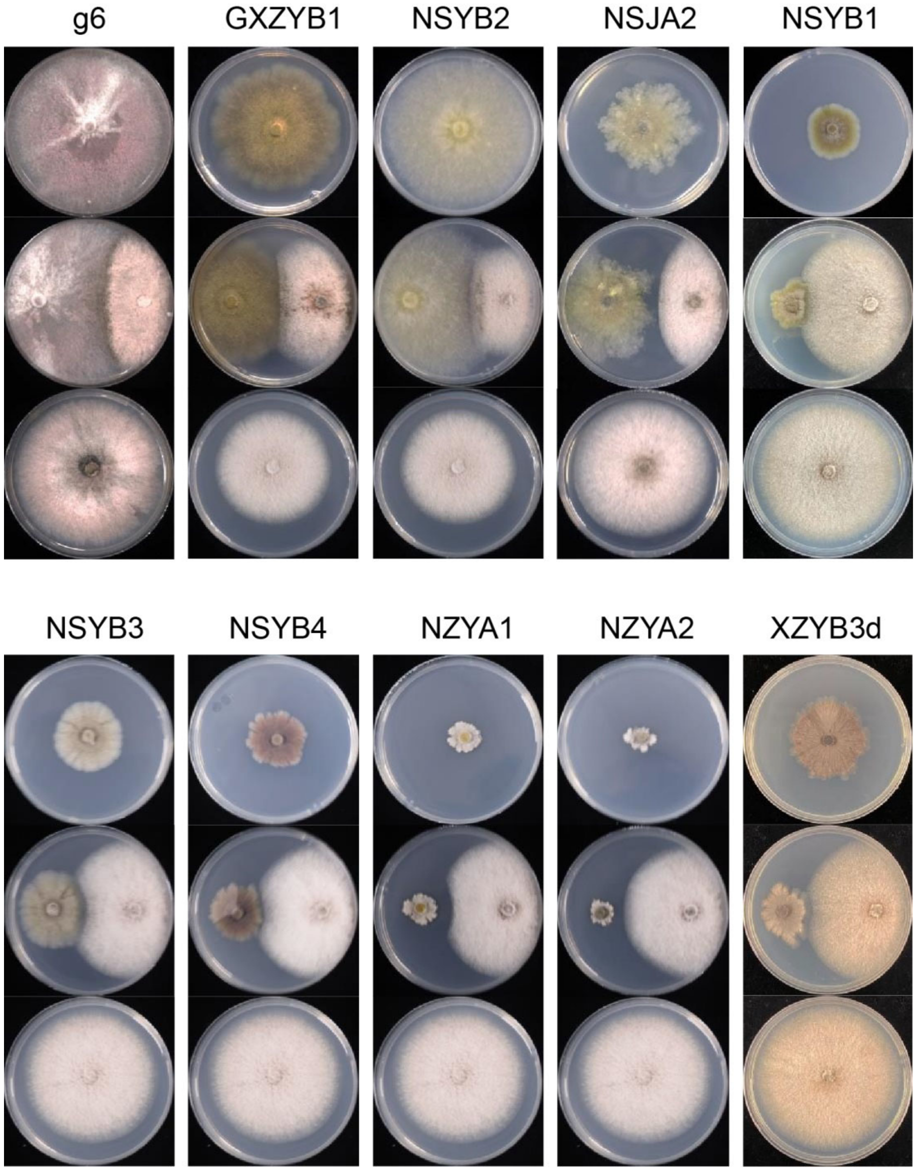

Fig. S9. Ten *Chaetomiaceae* isolates dual culture with *Lasiodiplodia theobromae* CSS-01S. The top, middle, and bottom three colonies in each part of the figure are: *Chaetomiaceae* isolates cultured separately, *Chaetomiaceae* isolates with CSS-01S dual culture, and CSS-01S cultured separately.

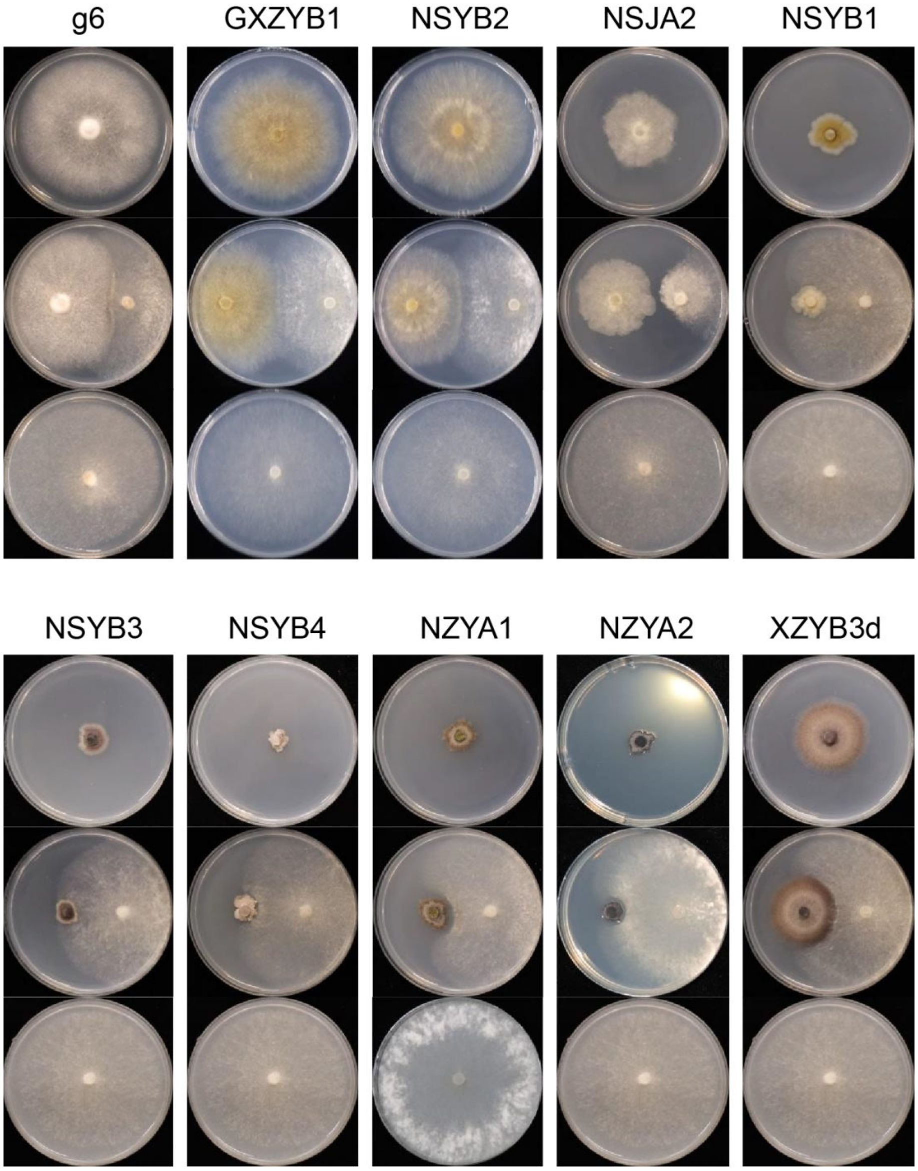

Fig. S10. Ten *Chaetomiaceae* isolates dual culture with *Sclerotinia minor* HJ5. The top, middle, and bottom three colonies in each part of the figure are: *Chaetomiaceae* isolates cultured separately, *Chaetomiaceae* isolates with HJ5 dual culture, and HJ5 cultured separately.

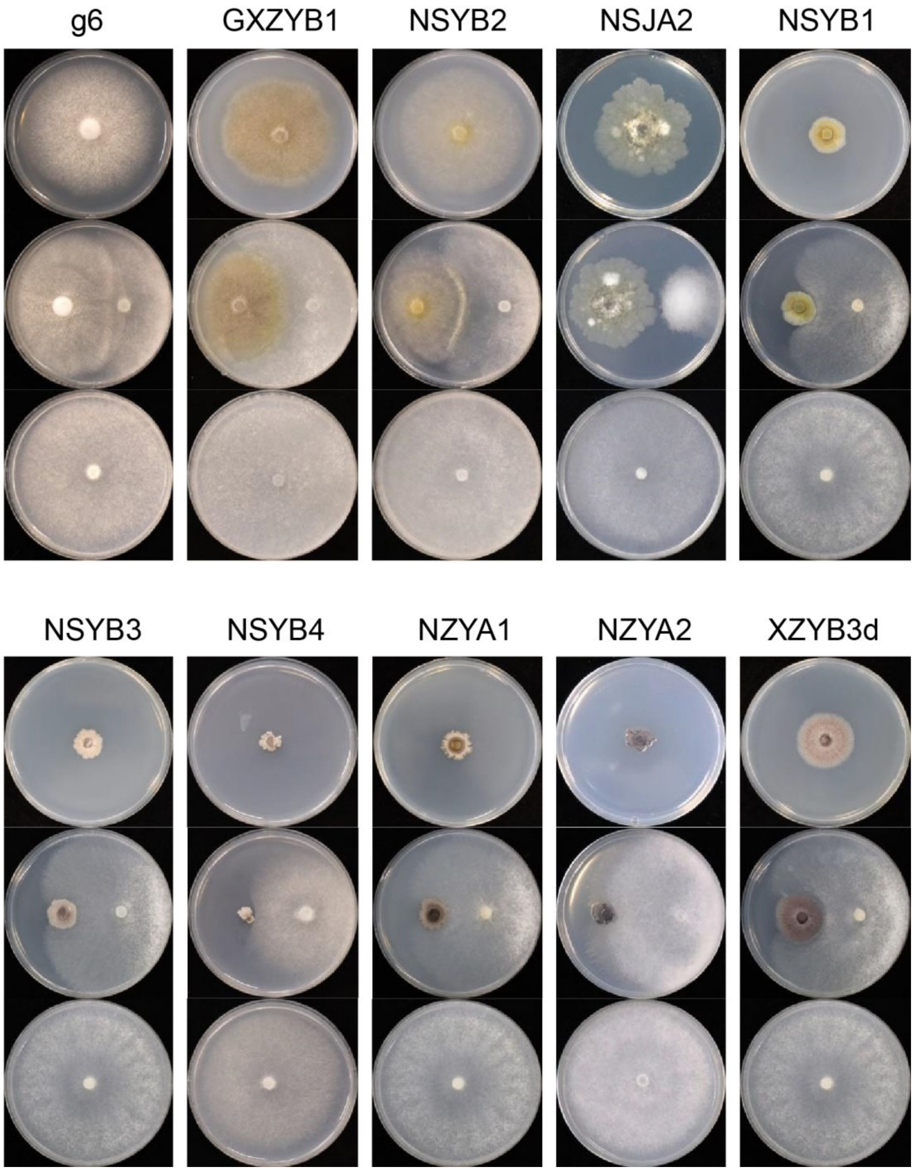

Fig. S11. Ten *Chaetomiaceae* isolates dual culture with *Sclerotinia sclerotiorum* ZJ25. The top, middle, and bottom three colonies in each part of the figure are: *Chaetomiaceae* isolates cultured separately, *Chaetomiaceae* isolates with ZJ25 dual culture, and ZJ25 cultured separately.

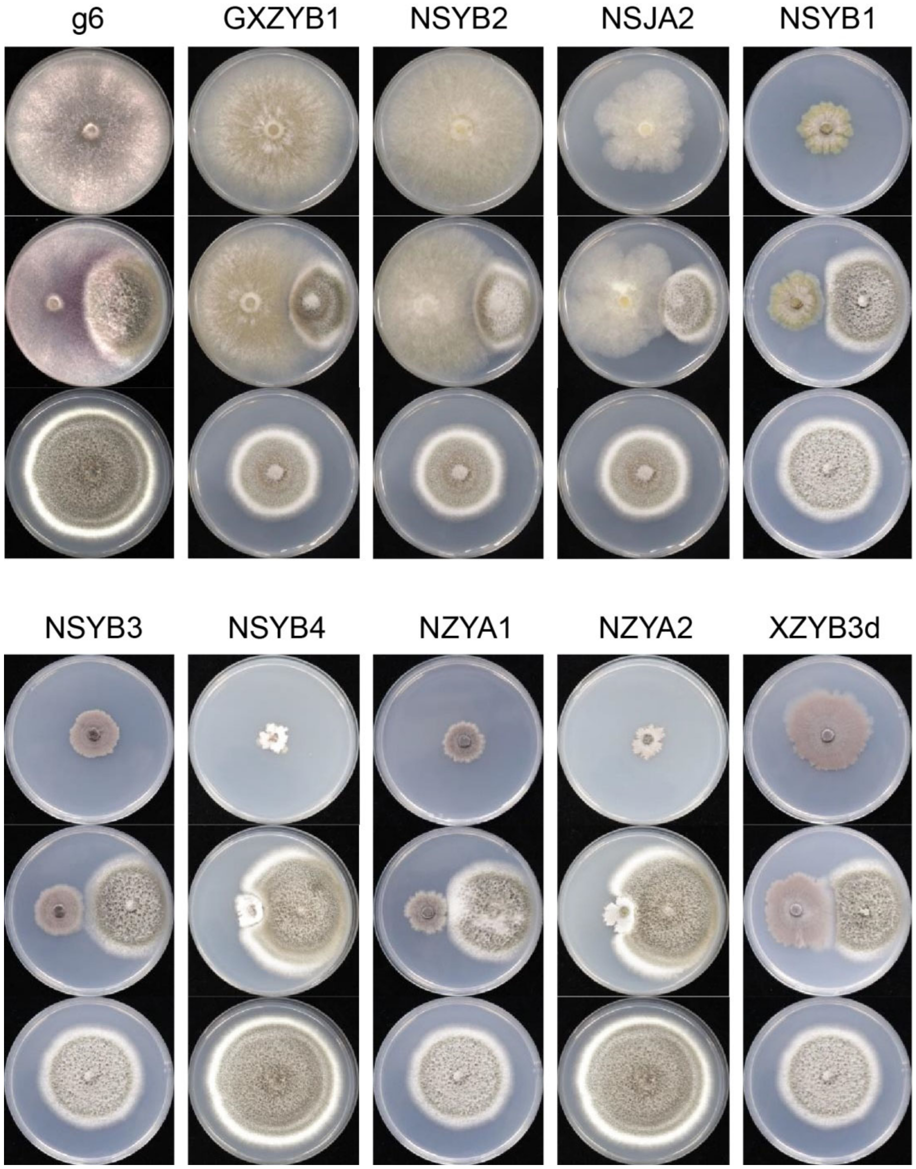

Fig. S12. Ten *Chaetomiaceae* isolates dual culture with *Stemphylium astragali* XZYB6f. The top, middle, and bottom three colonies in each part of the figure are: *Chaetomiaceae* isolates cultured separately, *Chaetomiaceae* isolates with XWYB6f dual culture, and XZYB6f cultured separately.

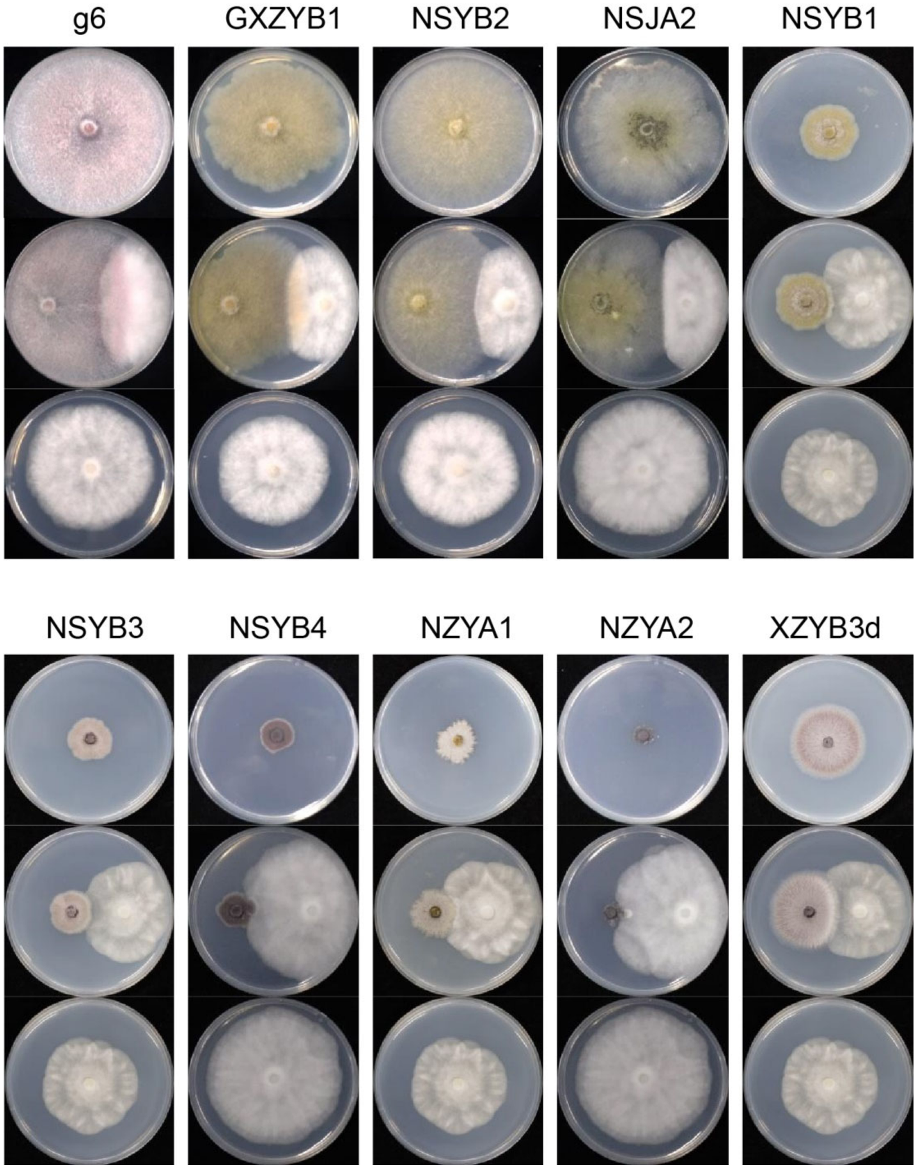

Fig. S13. Ten *Chaetomiaceae* isolates dual culture with *Phytophthora capsici* LT263. The top, middle, and bottom three colonies in each part of the figure are: *Chaetomiaceae* isolates cultured separately, *Chaetomiaceae* isolates with LT263 dual culture, and LT263 cultured separately.

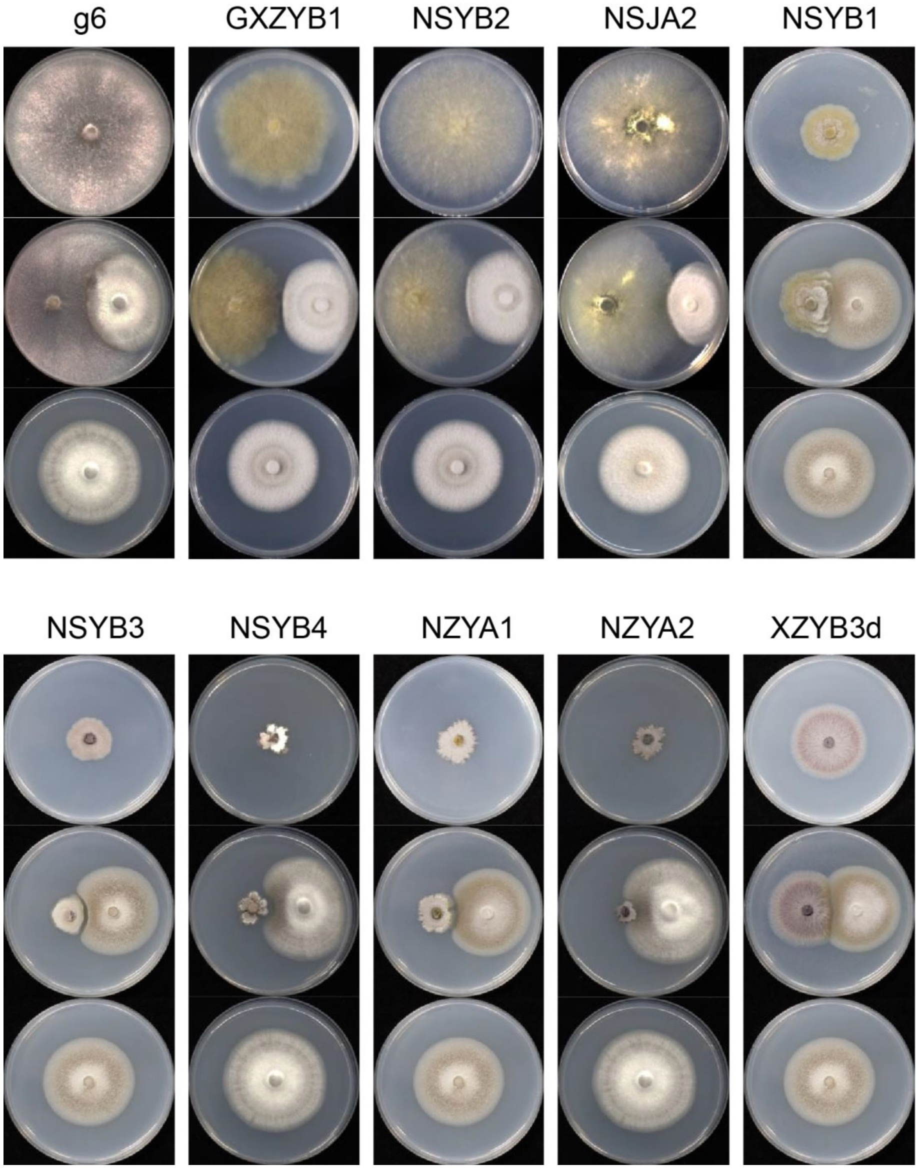

Fig. S14. Ten *Chaetomiaceae* isolates dual culture with *Pyricularia oryzae* P131. The top, middle, and bottom three colonies in each part of the figure are: *Chaetomiaceae* isolates cultured separately, *Chaetomiaceae* isolates with P131 dual culture, and P131 cultured separately.

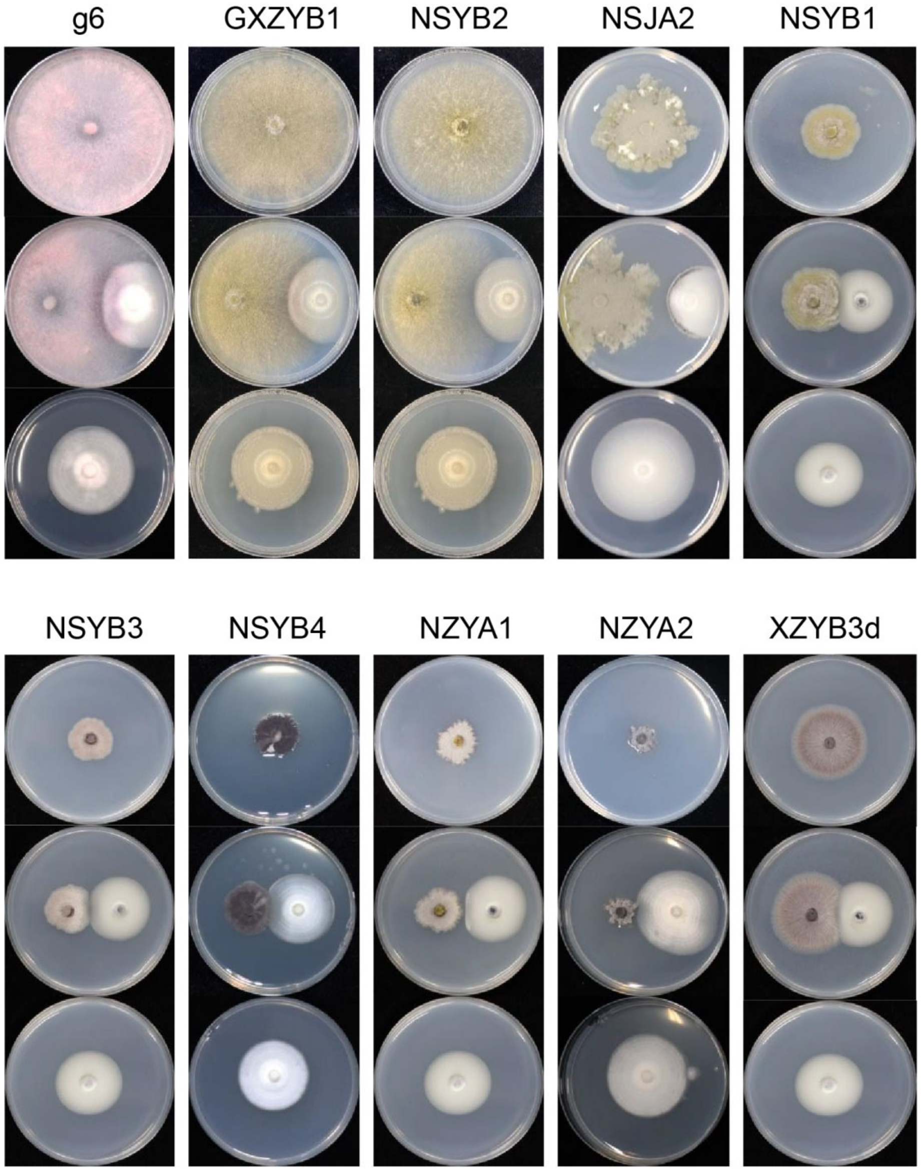

Fig. S15. Ten *Chaetomiaceae* isolates dual culture with *Verticillium dahlia* V991. The top, middle, and bottom three colonies in each part of the figure are: *Chaetomiaceae* isolates cultured separately, *Chaetomiaceae* isolates with V991 dual culture, and V991 cultured separately.

Table S1. Details of the reference sequence in this study.

Species	strain	GenBank accession numbers[a]			
		ITS	LSU	*rpb2*	*tub2*
Achaetomium aegilopis	IRAN 3453C T[b]	MT568841	MT568844	-	MT568852
A. globosum	CBS 332.67 T	KX976570	KX976695	KX976793	KX976911
A. lippiae	URM 7547 T	KY855413	KY855414	-	KY855412
A. luteum	CBS 618.68 R	KX976571	KX976696	KX976794	KX976912
A. macrosporum	CBS 152.97 R	KX976573	KX976698	KX976796	KX976914
A. strumarium	CBS 333.67 T	AY681204	AY681170	KC503254	AY681238
Botryotrichum atrogriseum	CBS 130.28 T	KX976589	KX976714	KX976813	KX976931
B. domesticum	UAMH 11929 T	MH899168	MH899169	MH899171	MH899172
B. foricae	CCF 5752 T	LR584032	LR584033	-	LR584034
B. geniculatum	CBS 144475 T	MZ334719	MZ351422	MZ342972	MZ343011
B. inquinatum	CBS 155.80 T	MK919289	MK919289	MK919345	MK919403
B. iranicum	ABRIICC 10152 T	MN134583	MN134576	MN128437	MN128435
B. murorum	CBS 163.52 R	KX976591	KX976716	KX976815	KX976933
B. murorum	CBS 173.68	KX976592	KX976717	KX976816	KX976934
B. peruvianum	CBS 460.90 R	KX976595	KX976720	KX976819	KX976937
B. piluliferum	CBS 654.79 T	KX976597	KX976722	KX976821	KX976939
B. retardatum	CBS 197.84 T	-	-	MZ342980	MZ343019
B. spirotrichum	CBS 211.55 T	KX976601	KX976726	KX976825	KX976943
B. trichorobustum	CBS 563.67 T	-	MZ351420	MZ342988	MZ343027
B. verrucosum	CBS 116.64 T	LT993567	LT993567	LT993486	LT993648
B. vitellinum	CBS 180.84 T	MZ334725	MZ351421	MZ342979	MZ343018
Chaetomium afropilosum	CBS 145.38 T	KT214574	KT214605	KT214675	KT214751
C. angustispirale	CBS 137.58 T	JX280764	JX280663	KF001824	JN256141
C. ascotrichoides	CBS 113.83 T	KC109752	KC109752	KF001832	KC109770
C. capillare	CBS 128489 T	KT214583	KT214614	KT214686	KT214760
C. cervicicola	CBS 128492 T	KT214558	KT214592	KT214662	KT214735
C. cirrhatum	CGMCC 3.17540 T	KP336792	KP336841	KT149508	KP336890
C. citrinum	CBS 693.82 T	KT214587	KT214617	KT214691	KT214764
C. coarctatum	CBS 162.62 T	JN209863	JN209863	KF001802	JN256142
C. cochliodes	CBS 155.52 ET	KC109754	KC109754	KF001811	KC109772
C. contagiosum	CBS 128494 T	KT214555	KT214589	KT214659	KT214732
C. cruentum	CBS 371.66 T	JN209871	JN209871	KF001795	JN256148
C. cucumericola	CBS 378.71 T	KT214579	KT214610	KT214680	KT214756
C. elatum	CBS 142034 NT	KX976612	KX976733	KX976832	KX976954
C. fimeti	CBS 139034 ET	KT214559	KT214593	KT214663	KT214736
C. globosum	CBS 160.62 NT	KT214565	KT214596	KT214666	KT214742
C. graminiforme	CBS 506.84 T	KT214584	KT214615	KT214687	KT214761
C. grande	CBS 126780 T	HM365253	HM365253	KT214657	HM365273
C. interruptum	CBS 126660 T	HM365246	HM365246	KT214665	KT214741
C. iranicum	IRAN 3379C T	-	-	MT273944	MN520421
C. madrasense	CBS 315.74 T	KC109751	KC109751	KF001831	KC109769
C. megalocarpum	CBS 149.59 ET	KC109744	KC109744	KF001828	KC109762
C. microthecium	CGMCC 3.17556 T	KP336785	KP336834	KT149505	KP336883

Species	strain	GenBank accession numbers[a]			
		ITS	LSU	*rpb2*	*tub2*
C. neoglobosporum	CBS 108.83 T	KC109750	KC109750	KF001825	KC109768
C. nepalense	CBS 288.83 T	MH861591	MH873316	MZ342983	-
C. novozelandicum	CBS 124555 T	KT214576	KT214607	KT214677	KT214753
C. nozdrenkoae	CBS 163.62 T	KT214556	KT214590	KT214660	KT214733
C. olivaceum	CBS 418.80A R	JN209914	JN209914	KF001806	JN256184
C. pilosum	CBS 335.67 T	KT214586	FJ666356	FJ666387	KT214763
C. pseudocochliodes	CGMCC 3.9441 T	JN209925	JN209925	KF001816	JN256195
C. pseudoglobosum	CBS 574.71 T	KT214573	KT214604	KT214674	KT214750
C. rectangulare	CBS 126778 T	HM365239	HM365239	KT214688	HM365285
C. spiculipilium	CBS 373.66 T	KC109756	KC109756	KF001809	KC109774
C. spirochaete	CBS 730.84 ET	JN209921	JN209921	KF001819	JN256191
C. subaffine	CBS 637.91 T	JN209929	JN209929	KF001817	JN256199
C. subfimeti	CBS 370.66 T	KT214562	FJ666354	FJ666385	KT214739
C. subglobosum	CBS 149.60 T	JN209930	JN209930	KF001808	JN256200
C. tarraconense	CBS 101882 T	-	-	MZ342964	MZ343005
C. tectifimeti	CBS 142032 T	KX976640	KX976737	KX976836	KX976982
C. telluricola	CBS 151.59 T	KT214582	KT214613	KT214685	KT214759
C. tenue	CBS 139.38 T	KT214568	KT214599	KT214669	KT214745
C. umbonatum	CBS 293.83 T	KT214575	KT214606	KT214676	KT214752
C. undulatulum	CBS 126775 T	HM365251	HM365251	KT214682	HM365279
C. unguicola	CBS 128446 T	KT214567	KT214598	KT214668	KT214744
Collariella anguipilia	CBS 632.83 T	MZ334721	MZ351424	MZ342989	MZ343028
Co. bostrychodes	CBS 163.73 R	KX976641	KX976738	KX976837	KX976983
Co. carteri	CBS 128.85 T	KX976647	KX976742	KX976841	KX976989
Co. causiiformis	CBS 792.83 T	KX976646	KX976741	KX976840	KX976988
Co. hexagonospora	CBS 171.84 T	MH861717	-	MZ342977	MZ343016
Co. hilkhuijsenii	CBS 143305 T	MG432011	MG432012	MF716587	MF716586
Co. pachypodioides	CBS 164.52 T	MH856980	MH868500	MZ342975	MZ343014
Co. quadrangulata	CBS 152.59 R	KX976651	KX976746	KX976845	KX976993
Co. quadrum	CGMCC 3.17917 T	KU746675	KU746721	KY575870	KU746767
Co. robusta	CBS 551.83 T	KX976652	KX976747	KX976846	KX976994
Subramaniula anamorphosa	CBS 137114 T	KP862598	KP970641	KP900667	KP900704
S. asteroides	CBS 123294 T	HQ906667	JX280731	KP900666	KP900703
S. cristata	CBS 156.52 T	KX976690	KX976788	KX976903	KX977038
S. cristata	DTO 324-H7	KX976691	KX976789	KX976904	KX977039
S. cuniculorum	CBS 800.83 R	KX976692	KX976790	KX976905	KX977040
S. cuniculorum	CBS 121.57	KP862602	KP970644	KP900671	KP900709
S. flavipila	CBS 446.66 T	KP862600	KP970647	KP900669	KP900706
S. fusispora	CBS 166.61 T	MH858011	MH869571	MZ342976	MZ343015
S. lateralis	CGMCC 3.17547 T	KP336789	KP336838	MZ342998	KP336887
S. latifusispora	CGMCC 3.20442	MZ334728	MZ351428	MZ343001	MZ343040
S. obscura	CBS 132916 T	KP862595	KP970653	KP900662	KP900700
S. thielavioides	CBS 122.78 T	KP862597	KP970654	KP900670	KP900708
Condenascus tortuosus	CBS 610.97 R	MK926817	MK926817	MK876777	MK926917

[a] Sequences generated in this study are indicated in bold.

[b] T, ET and NT indicate ex-type, ex-epitype and ex-neotype strains, respectively.

Table S2.Radial inhibited rate (%) of phytopathogens in dual culture

Phytopat-ogens	10 isolates of *Chaetomiaceae*				
	g6	GXZYB1	NSYB2	NSJA2	NSYB1
F0609	33.73±1.36 c[a]	50.21±2.55 b	28.94±3.90 c	61.60±6.37 a	20.25±25.32 d
B79	69.53±2.97 ab	67.53±2.60 bc	72.73 a	64.07±1.50 c	36.36 e
CCT1	51.40±3.35 bc	67.03±3.75 a	63.78±1.87 a	54.55±4.90 b	32.09±1.85 d
Foc	38.30 c	46.51 b	34.11±2.69 d	58.06 a	25.85±4.71 e
XZYB6f	53.47±0.86 c	61.44±2.26 b	61.44±2.26 b	70.59±3.92 a	33.96 e
HJ5	83.80±1.60 a	38.77±1.53 b	37.89±2.64 b	81.01±2.53 a	12.99±6.87 d
ZJ25	85.90±2.56 a	57.25±2.72 c	56.47±23.53 c	79.84±1.43 b	56.47±2.35 c
P131	34.72±3.18 e	14.81±2.57 f	11.85±2.57 f	56.82 ab	52.38±2.38 bc
B-8-1	51.03±1.43 c	83.12±3.44 a	87.45±1.50 a	64.94±2.60 b	52.81±5.41 c
LT263	72.25±1.81 a	46.78±2.03 b	47.95± 2.03 b	76.33±3.35 a	26.12±8,96 d
V991	28.83±3.12 bc	18.25±2.75 de	23.02±2.75 cd	59.18 a	35.29±4.08 b
HEYA2	44.44±3.50 b	32.52±7.45 c	22.76±28.16 cd	67.47±12.52 a	13.25±3.61 d
A33	70.20±3.97 a	65.90±2.00 a	68.51 a	65.19 a	35.36±5.74 bc
Y3-3	7.41 d	57.66±17.38 a	5.41 d	59.42±2.51 a	31.88±2.51 b
CSS-01S	45.33±2.67 b	29.83±1.91 c	44.03±4.36 b	54.27±4.61 a	18.67 d
	NSYB3	NSYB4	NZYA1	NZYA2	XZYB3d
F0609	50.63±25.32 b	18.57±1.46 d	3.38±2.92 e	6.33±3.80 e	23.08±2.56 d
B79	17.32±3.00 h	19.05±3.00 gh	31.17±2.60 f	22.51±3.97 g	42.42±3.97 d
CCT1	22.46±1.85 e	17.11±4.90 e	28.88±1.85 d	21.39±5.56 e	45.99±1.85 c
Foc	10.88±4.71 g	4.08 h	20.41 f	16.33 f	17.69±2.36 f
XZYB6f	15.09 g	15.35 g	13.84±2.18 g	19.80±2.57 f	41.51 d

HJ5	13.85±9.83 d	18.18±2.60 d	25.97±4.50 c	10.98±1.36 d	15.58±2.60 d
ZJ25	11.76 f	23.38±2.60 e	29.02±5.92 d	16.47±2.35 f	25.88±2.35 de
P131	46.83±3.64 cd	14.41±3.12 f	41.27±5.50 d	10.81±5.41 f	59.52±4.76 a
B-8-1	54.55±6.87 c	30.38 d	2.60 f	14.35±1.46 e	60.61±1.50 b
LT263	33.33±2.22 c	10.26±1.78 e	32.59±3.40 cd	5.13±1.78 e	49.63±4.63 b
V991	9.41±2.04 f	20.20±3.50 d	16.47±5.39 de	13.01±2.82 ef	32.94±6.11 b
HEYA2	18.07±2.09 d	24.39±4.88 cd	14.46±5.52 d	16.33 d	21.69±7.52 cd
A33	27.62±5.06 d	34.48±2.39 c	37.57±3.83 bc	2.35±4.14 d	40.88±1.91 b
Y3-3	33.33±2.51 b	7.03±2.71 d	33.33±6.24 b	12.58±2.18 cd	20.29±6.64 c
CSS-01S	30.92±1.67 c	22.22±4.42 d	18.36±1.67 d	4.83±1.67 f	13.12±1.36 e

[a]Lowercase letters represent significant differences between treatments ($p < 0.05$).

Table S3.Radial inhibited rate (%) of 10 isolates of *Chaetomiaceae* in dual culture

Phytopath-	10 isolates of *Chaetomiaceae*				
ogens	g6	GXZYB1	NSYB2	NSJA2	NSYB1
F0609	80.17±1.46 a[a]	42.11 c	67.07±5.49 b	5.41 e	17.39 d
B79	37.67±3.11 a	29.44±1.76 b	37.56±1.63 a	11.20 d	27.85 b
CCT1	53.16±2.53 a	32.86±1.63 d	44.65±1.61 b	29.79 e	17.58 g
Foc	58.23 a	35.75±1.67 b	32.84 bc	4.44 g	34.57±4.28 b
XZYB6f	21.96±1.36 b	20.60±2.97 b	20.39±1.36 b	6.99±2.42 c	8.00 c
HJ5	28.64 cd	25.12±4.43 d	29.15±3.02 cd	6.67±1.65 e	36.00±6.93 bc
ZJ25	15.71 d	33.89±8.39 b	22.22 c	8.16 e	15.22 d
P131	35.29 bc	31.02 c	36.77 bc	18.67±2.67 d	37.18±2.22 bc
B-8-1	52.05 a	29.81±4.30 bc	35.90±8.88 b	9.21 e	15.22 d
LT263	23.52 bc	17.91 cd	35.39±2.85 a	16.89±1.54 cd	8.97±2.22 e
V991	25.88 cd	32.03±3.00 bc	35.29±2.35 b	11.26±2.37 gh	24.36±2.22 de
HEYA2	34.51±1.36 b	23.38±2.60 c	38.43±2.72 b	13.27±1.53 d	38.96±2.25 b
A33	52.49 b	62.75 a	62.91±1.63 a	9.68±2.79 e	35.82±10.34 c
Y3-3	55.69±1.36 a	28.69±3.87 c	21.72±1.57 d	6.51 e	21.11±1.93 d
CSS-01S	32.16±2.71 b	27.60±1.80 b	20.25±2.53 c	14.29±2.04 de	19.67±5.68 c
	NSYB3	NSYB4	NZYA1	NZYA2	XZYB3d
F0609	18.18 d	11.76 de	37.74 c	13.79±11.95 d	35.79±3.65 c
B79	16.39 c	10.53 d	18.18 c	31.43±8.57 b	28.17 b
CCT1	21.54 f	23.88 f	21.54 f	15.38 g	35.77±2.82 c
Foc	9.84±5.68 f	18.18 e	24.56±6.07 d	28.57 cd	13.79 ef
XZYB6f	8.70 c	18.92±8.11 b	11.76 c	27.78±4.81 a	21.71±2.69 b

HJ5	48.48±10.50 a	28.57 cd	30.56±9.62 cd	44.44 ab	22.52±4.13 d
ZJ25	38.24 b	57.14 a	25.00 c	37.50 b	26.67±4.62 c
P131	42.11±9.12 b	16.67 d	29.82±8.04 c	18.18 d	54.05±5.41 a
B-8-1	20.59 d	27.03 c	20.59 d	50.00 a	34.67 b
LT263	21.05 bcd	23.53±5.09 bc	14.04±6.08 de	23.81±8.25 bc	27.03±2.70 b
V991	7.02±3.04 h	21.05 def	17.54±3.04 efg	15.38 fg	44.14±10.92 a
HEYA2	57.14±5.36 a	21.05 c	40.00±5.46 b	41.03±8.89 b	55.05±1.59 a
A33	54.24 b	24.07±6.41 d	40.00±6.67 c	18.18 d	52.87±3.98 b
Y3-3	29.51±5.68 c	38.89 b	57.58±2.62 a	41.67 b	33.61±4.92 c
CSS-01S	10.31 e	19.28±4.17 cd	18.92 cd	14.29 de	47.79±3.07 a

[a] Lowercase letters represent significant differences between treatments ($p < 0.05$).
